# Calpain-mediated proteolysis as driver and modulator of polyglutamine toxicity

**DOI:** 10.3389/fnmol.2022.1020104

**Published:** 2022-10-19

**Authors:** Rana Dilara Incebacak Eltemur, Huu Phuc Nguyen, Jonasz Jeremiasz Weber

**Affiliations:** ^1^Department of Human Genetics, Ruhr University Bochum, Bochum, Germany; ^2^Institute of Medical Genetics and Applied Genomics, University of Tübingen, Tübingen, Germany

**Keywords:** posttranslational modifications (PTMs), proteolytic cleavage, calpains, toxic fragments, Huntington disease (HD), spinocerebellar ataxia (SCA), dentatorubral-pallidoluysian atrophy (DRPLA), spinal and bulbar muscular atrophy (SBMA)

## Abstract

Among posttranslational modifications, directed proteolytic processes have the strongest impact on protein integrity. They are executed by a variety of cellular machineries and lead to a wide range of molecular consequences. Compared to other forms of proteolytic enzymes, the class of calcium-activated calpains is considered as modulator proteases due to their limited proteolytic activity, which changes the structure and function of their target substrates. In the context of neurodegeneration and - in particular - polyglutamine disorders, proteolytic events have been linked to modulatory effects on the molecular pathogenesis by generating harmful breakdown products of disease proteins. These findings led to the formulation of the *toxic fragment hypothesis*, and calpains appeared to be one of the key players and auspicious therapeutic targets in Huntington disease and Machado Joseph disease. This review provides a current survey of the role of calpains in proteolytic processes found in polyglutamine disorders. Together with insights into general concepts behind *toxic fragments* and findings in polyglutamine disorders, this work aims to inspire researchers to broaden and deepen the knowledge in this field, which will help to evaluate calpain-mediated proteolysis as a unifying and therapeutically targetable posttranslational mechanism in neurodegeneration.

## Introduction

One of the most impactful posttranslational modifications (PTMs) is proteolytic fragmentation, a process in which proteases cleave and trim their target proteins, thereby directly affecting the structural integrity of the substrate. These changes, which range from removal of single amino acids up to ablation of entire domains, may have major consequences on the function, localization, interactome, and stability of the affected protein, including the availability of sequences for further PTMs. In comparison to other modifications such as phosphorylation, lipidation, or ubiquitination, proteolytic fragmentation is an irreversible process, strongly determining the fate of its substrate. The field of research, which deals with the entirety of these proteolytic processes, both in physiological and pathological contexts is termed degradomics (Rogers and Overall, 2013; Klein et al., 2018). In a more pathological context, proteolytic fragmentation can lead to the generation of detrimental protein species, which might harm affected cells and organs and culminate in medical conditions, as highlighted by the secretase-based processing of amyloid precursor protein (APP) in Alzheimer’s disease (AD; Selkoe and Hardy, 2016). In a further context of neurodegeneration, proteolytic fragmentation of disease proteins in polyglutamine (polyQ) disorders has been identified as pivotal modulators of their molecular pathology (Wellington and Hayden, 1997). Multiple proteases were associated with polyQ protein fragmentation, including apoptosis-associated caspases, lysosomal cathepsins, as well as matrix metalloproteinases. Calpains, a class of calcium-activated proteolytic enzymes, emerged as one of the key players in polyQ disorders (Weber et al., 2014; Matos et al., 2017). Here, we provide a comprehensive overview of the current knowledge on the involvement of calpains in this group of rare and yet incurable diseases, emphasizing the relevance of further investigations for improving our understanding of calpain-mediated cleavage and, thereby, uncovering new therapeutic avenues for neurodegeneration.

## Calpains

Calpains are a family of calcium-dependent, intracellular cysteine proteases implicated in the maintenance of cellular homeostasis and a variety of physiological processes (Ono and Sorimachi, 2012). Their initial discovery can be traced back to the early 1960s when the first calcium-activated neutral proteinase was extracted from rat brain (Guroff, 1964). Several years later, this class of proteases was collectively renamed calpains - combining “cal” as an abbreviation for calcium and reference to the calcium-binding protein calmodulin, with “pain” in a nod to structurally similar cysteine proteases like papain (Murachi et al., 1980). Calpains and their respective homologs are an evolutionarily distinct group of proteases and are found in nearly all unicellular and multicellular eukaryotes, including animals, fungi, and plants, as well as in some bacteria but not archaea (Goll et al., 2003; Sorimachi et al., 2011; Ono and Sorimachi, 2012). The highest diversity of calpain homologs can be found in animals, with the largest number of 15 different enzymes present in mammals, and thus also in the human genome. This circumstance has enabled researchers to investigate the physiological and pathophysiological roles of human calpains in classical rodent models such as mice and rats, being the foundation of a vast number of significant studies in the field (Goll et al., 2003; Sorimachi et al., 2011; Ono and Sorimachi, 2012).

**Figure 1:**
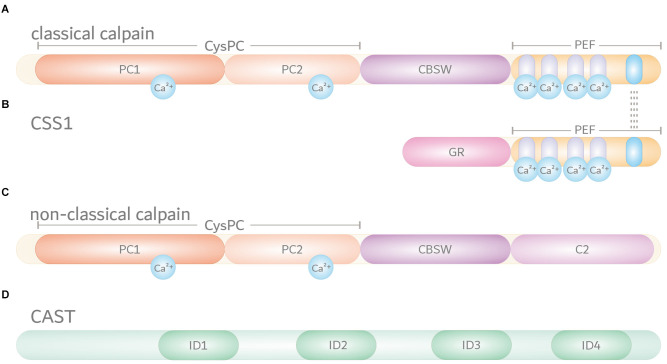
Domain composition of the members of the intracellular calpain system. Based on their domain composition, calpains are divided into two main groups. **(A)** Classical calpains, including the conventional calpains calpain-1 and calpain-2, are characterized by a proteolytically active CysPc domain harboring two core domains PC1 and PC2. In addition, classical calpains comprise a calpain-type β-sandwich (CBSW) domain and a C-terminal calcium-binding penta-EF-hand (PEF) domain, through which they form inactive stable heterodimers with the regulatory calpain small subunit 1 (CSS1). Individual EF-hand motifs within the PEF domain are illustrated by light purple- and blue-shaded ovals. Interaction with CSS1 via the fifth EF-hand motif is highlighted by dashed lines. Binding of calcium ions (Ca^2+^) in their respective numbers along the protein structure is indicated by cyan-colored circles. **(B)** The CSS1 consists of a glycine-rich region (GR) and a PEF domain through which interaction with conventional calpains occurs. **(C)** Non-classical calpains come in a variety of structural and domain compositions. Aside from sharing the CysPc domain as a common trait, most of them comprise a CBSW domain and, for example, a C-terminal C2 domain. **(D)** Endogenous calpain inhibitor calpastatin (CAST) contains four repetitive inhibitory domains (ID) that bind to CysPc and PEF domains in calpains, thereby inhibiting their proteolytic function.

Calpains are primarily characterized by their highly conserved calpain-like cysteine protease domain (CysPc), which can be divided into the two core domains PC1 and PC2. Both core units bind one calcium ion each, leading to their conformational rearrangements and activation of the catalytic triad composed of cysteine, histidine, and asparagine residues (Arthur et al., 1995; Moldoveanu et al., 2002). Most calpains feature one central calpain-type β-sandwich (CBSW) domain with calcium-binding properties, followed by an additional C-terminal domain (Tompa et al., 2001; Ono et al., 2016). Based on their structural and domain-wise composition, the known 15 members of the calpain family in humans can be grouped into classical (calpain-1, -2, -3, -8, -9, 11–14) or non-classical calpains (calpain-5, -6, -7, -10, -15, -16; Table 1; Sorimachi et al., 2011; Ono and Sorimachi, 2012). Classical calpains share common structural features, comprising - aside from the aforementioned CysPc domain - a C-terminal penta-EF-hand (PEF) domain with five identical calcium-binding EF-hand motifs, of which four bind calcium ions (Figure 1A; Table 1; Blanchard et al., 1997; Lin et al., 1997). The fifth EF-hand motif is essential for the so-called conventional calpains, calpain-1 and calpain-2, to form inactive stable heterodimers with the regulatory calpain small subunit 1 (CSS1, earlier known as calpain-4). CSS1 is composed of an N-terminal glycine-rich region (GR) and a C-terminal PEF domain which, corresponding to the PEF domain in the large catalytic subunits, also binds four calcium ions and facilitates the interaction with conventional calpains (Figure 1B). It has been reported that, as a part of the calcium-dependent calpain activation process, CSS1 may dissociate from the large subunits. However, other studies suggested that CSS1 remains in the dimer when activated (Ravulapalli et al., 2009; Sorimachi et al., 2011; Ono and Sorimachi, 2012). Interestingly, knockout of CSS1 resulted in destabilization and activity loss of calpain-1 and calpain-2, and was found to result in embryonic lethality (Arthur et al., 2000; Zimmerman et al., 2000). Although all classical calpains are characterized by their C-terminal PEF domain, the function of CSS1 and interaction with other calpains remain unclear (Ono and Sorimachi, 2012). Moreover, the existence of a second small regulatory subunit (CSS2) is known, but its functions are even less understood (Schád et al., 2002; Ma et al., 2004). Unlike classical calpains, non-classical calpains appear in various structural compositions, and lack or have a replacement for typical domains like the PEF domain (Figure 1C, Table 1; Sorimachi et al., 2011; Ono and Sorimachi, 2012; Ono et al., 2016; Nian and Ma, 2021; Spinozzi et al., 2021).

**Table 1:** Protein structure and expression preference of calpain system-related genes and associated clinical conditions.

**Gene (Protein)**	**Structural composition**	**Expression preference**	**Linked clinical conditions**	References*
**Classical calpains**				
*CAPN1* (calpain-1)		ubiquitous	SPG76^c^, cerebellar ataxia^c^	Gan-Or et al. (2016) and Wang et al. (2016)
*CAPN*2 (calpain-2)		ubiquitous, except erythrocytes	embryonic lethality^e^	Takano et al. (2011)
*CAPN3* (calpain-3)		skeletal muscle	LGMDR1^c^, LGMDD4^c^	Richard et al. (1995) and Vissing et al. (2016)
*CAPN8* (calpain-8)		gastrointestinal tract	Gastric ulcer^e^	Hata et al. (2010)
*CAPN9* (calpain-9)	CysPC (PC1, PC2); CBSW, PEF	gastrointestinal tract	Gastric ulcer^e^	Hata et al. (2010)
*CAPN11* (calpain-11)		testis	n/a	n/a
*CAPN12* (calpain-12)		hair follicle	Congenital ichthyosis^a^	Bochner et al. (2017)
*CAPN13* (calpain-13)		ubiquitous	n/a	n/a
*CAPN14* (calpain-14)		ubiquitous	Eosinophilic oesophagitis^s^	Kottyan et al. (2014)
**Non-classical calpains**				
*CAPN5* (calpain-5)		ubiquitous	ADNIV^c^	Mahajan et al. (2012)
*CAPN6* (calpain-6)	MIT; CysPC (PC1, PC2); CBSW; CBSW/C2	embryonic muscles, placenta	n/a	n/a
*CAPN7* (calpain-7)		ubiquitous	n/a	n/a
*CAPN10* (calpain-10)		ubiquitous	Type 2 diabetes mellitus^s^	Horikawa et al. (2000)
*CAPN15* (calpain-15)	Zn, CysPC (PC1, PC2); SOH	ubiquitous	OGIN^c^	Zha et al. (2020)
*CAPN16* (calpain-16)	PC1; IQ	ubiquitous	n/a	n/a
**Other members of the calpain system**				
*CAPNS1* (CSS1)	GR; PEF	ubiquitous	embryonic lethality^e^	Arthur et al. (2000) and Zimmerman et al. (2000)
*CAPNS2* (CSS2)	PEF	ubiquitous	n/a	n/a
*CAST* (CAST)	ID1; ID2; ID3; ID4	ubiquitous	PLACK^c^	Lin et al. (2015)

Summary of calpain system-related genes, structural composition and expression preference of encoded proteins, as well as clinical conditions linked to mutations and variants of these genes. Information on genes, structural composition, and expression were retrieved from Sorimachi et al. (2011), Ono and Sorimachi (2012), Sorimachi and Ono (2012), and Ono et al. (2016). Additional information from the OMIM^®^ database. ADNIV, autosomal dominant neovascular inflammatory vitreoretinopathy; CBSW, calpain-type β-sandwich; C2, C2 domain; C2L, C2-like domain; CSS1/2, calpain small subunit 1 or 2; CysPC, calpain-like cysteine protease domain; GR, glycine-rich region; ID, inhibitory domain; IQ, calmoduling-interacting motif; LGMDD4, autosomal dominant limb-girdle muscular dystrophy 4; LGMDR1, autosomal recessive limb-girdle muscular dystrophy 1; MIT, microtubule interacting and transport motif; n/a, not available; OGIN, oculogastrointestinal neurodevelopmental syndrome; PC, protease core domain; PEF, penta-EF-hand domain; PLACK, peeling skin with leukonychia, acral punctate keratoses, cheilitis, and knuckle pads; SPG76, spastic paraplegia 76; SOH, SOL-homology domain; Zn, Zinc finger motif. *Only references reporting on disease-causing or susceptibility associations. ^a^association; ^c^causation; ^e^causation, only experimental; ^s^susceptibility.

The expression of calpain family members ranges from wide distributions to very limited expression patterns. Whereas calpain-1, -2, -5, -7, -10, 13–16, and CSS1/2 are expressed ubiquitously (Sorimachi et al., 2011; Ono and Sorimachi, 2012), the remaining calpains are restricted to a tissue-specific expression (Table 1). For instance, calpain-3 or calpain-12 are solely expressed in skeletal muscle or hair follicle cells, respectively (Sorimachi et al., 1989; Dear et al., 2000).

The calpain system does not only include various calpain enzymes and their regulatory subunit but also calpastatin (CAST), the only endogenous and ubiquitously expressed inhibitor of classical calpains (Ono and Sorimachi, 2012). CAST features four repetitive inhibitor domains with distinct specificities, by which it can bind, amongst others, to the proteolytic CysPc and PEF domains, looping out and around the catalytically active cysteine residue, eventually inhibiting calpain activation (Figure 1D; Hanna et al., 2008; Moldoveanu et al., 2008).

Calpains are referred to as modulator proteases, as they do not randomly cleave and degrade proteins but perform a limited proteolysis on their substrates. More specifically, they remove distinct motifs and domains from their target proteins, thereby modulating their structure, function, and activity (Sorimachi et al., 2011; Ono et al., 2016). They participate in a variety of vital cellular processes, including cell cycle and proliferation, apoptosis, cell motility, signal transduction pathways (Goll et al., 2003), neurogenesis (Baudry et al., 2021), and synaptic plasticity (Baudry and Bi, 2016). Based on their wide spectrum of cellular functions, disruption, and dysregulation of calpains are associated with various diseases (Ono et al., 2016). Noteworthy, calpainopathies are specified as disorders explicitly caused by mutations in calpain genes. This family of disorders includes forms of limb-girdle muscular dystrophy and autosomal dominant neovascular inflammatory vitreoretinopathy (ADNIV), which are caused by mutations in calpain-3 and calpain-5, respectively (Gallardo et al., 2011; Mahajan et al., 2012; Vissing et al., 2016). For a comprehensive overview of diseases caused by, or linked to, mutations and variants in calpain system-related genes, see Table 1. Moreover, dysregulations of the intracellular calpain system are associated with neurodegenerative disorders, like AD, Parkinson’s disease (PD), amyotrophic lateral sclerosis (ALS), and polyglutamine (polyQ) disorders. Furthermore, calpains were shown to be implicated in other diseases, such as cancer, cardiovascular and ischemic disorders, and diabetes (Ono et al., 2016).

### Calpains and the toxic fragment hypothesis

Aberrant proteolytic processing of disease proteins by proteases is a phenomenon observed in several neurodegenerative disorders. About three decades ago, disease-associated proteolysis was described for the first time in AD (Esch et al., 1990; Selkoe, 1994; de Strooper and Annaert, 2000). Here, altered cleavage of APP by secretases results in amyloid beta peptides that form highly insoluble amyloid plaques and have neurotoxic properties (Esler and Wolfe, 2001; LaFerla et al., 2007; Selkoe and Hardy, 2016). Some years later, the so-called *toxic fragment hypothesis* extended the concept to polyQ disorders by postulating that polyQ stretch-containing fragments of disease proteins are more toxic than their full-length forms (Wellington and Hayden, 1997). Also, in synucleinopathies such as certain forms of Parkinson’s disease (PD) or, for instance, in SOD1- or TDP-43-linked ALS, disease protein cleavage was identified as a disease-modifying factor (Wright and Vissel, 2016; Bluhm et al., 2021; Chhangani et al., 2021). While protein-degrading machineries, such as autophagy and ubiquitin-proteasome system, lead to a full disintegration of proteins to amino acids or peptides, multiple classes of proteolytic enzymes, including caspases, cathepsins, matrix metalloproteinases, secretases, and calpains were associated with the production of disease protein fragments in polyQ disorders (Weber et al., 2014; Matos et al., 2017). Due to the still incomplete picture of their degree of involvement in disease protein cleavage, and thus in the molecular pathogenesis, a reasonable ranking of these proteases’ disease-modifying significance in the context of polyQ disorders is unattainable. However, it is noteworthy that calpains, due to their particular function as modulator proteases, were found to act upstream of proteolytic processes, controlling degradational mechanisms such as autophagy, and regulating caspase activation under apoptotic or degenerative conditions (Yousefi et al., 2006; Gafni et al., 2009; Smith and Schnellmann, 2012; Sorimachi and Ono, 2012; Weber et al., 2019a). Based on these considerations, calpains emerge as important modulators of disease protein toxicity, thus representing excellent targets for therapeutic intervention (Weber et al., 2014; Matos et al., 2017).

### Calpains in polyglutamine diseases

#### Polyglutamine diseases

Among the large group of inherited neurodegenerative conditions, the clinically and phenotypically heterogeneous class of polyQ disorders is defined by a common type of causative mutation, the expansion of an exonic CAG repeat motif in the affected gene. The CAG base triplet codes for the amino acid glutamine and is translated into an elongated polyQ stretch in the disease protein. So far, the family of polyQ disorders comprises nine rare conditions, namely the spinocerebellar ataxias 1 (SCA1), 2 (SCA2), 3 (SCA3; also known and hereinafter referred to as Machado-Joseph disease, MJD), 6 (SCA6), 7 (SCA7), and 17 (SCA17) as well as Huntington disease (HD), dentatorubral-pallidoluysian atrophy (DRPLA) and spinal and bulbar muscular atrophy (SBMA). The inheritance of these diseases is autosomal dominant, except for SBMA which follows an X-linked recessive pattern (Paulson et al., 2017; Stoyas and la Spada, 2018).

The clinical manifestation of the mutation depends on disease-specific thresholds of the highly polymorphic CAG repeat/polyQ lengths, with most diseases featuring a defined intermediate expansion range linked to reduced penetrance. Above that range, the disease shows full manifestation, with both age at onset (AAO) and symptomatic severity negatively correlating with expansion length. Due to an instability of the CAG repeat during meiosis, polyQ disorders are furthermore characterized by the genetic phenomenon of anticipation, leading to longer expansions and increased severity in the next generation (McMurray, 2010). Interestingly, the observed discrepancy in AAO cannot be explained by the CAG repeat length alone, suggesting additional genetic modifiers and environmental influences as major contributing factors (Chen et al., 2018). As their name implies, SCAs are primarily characterized by the occurrence of ataxic symptoms, triggered by the degeneration of the cerebellum and brainstem, whereas HD shows symptoms such as hyperkinetic movements (chorea) and neuropsychological manifestations, caused by damage in the striatum and cerebral cortex. DRPLA features ataxia, epilepsy, and intellectual deterioration based on atrophy of the cerebellum and brainstem, and SBMA is defined by a motoneuron loss-dependent muscle weakness and wasting. All polyQ disorders are highly impairing, life-shortening, and - at the present moment - incurable (Paulson et al., 2017; Stoyas and la Spada, 2018). These pathophysiological and neuropathological differences between polyQ disorders can be largely attributed to the diversity in affected genes and proteins they encode. The giant huntingtin protein (HTT) involved in HD was shown to serve as a scaffold protein for a large number of interacting partners, employing it in multiple pathways and mechanisms such as autophagy, cell division, endocytosis, vesicle trafficking, and transcriptional regulation. The SCA1 protein ataxin-1 is involved in transcriptional repression, a function it shares with the DRPLA protein atrophin-1. Ataxin-2 (Atx2), the disease protein of SCA2, which has been also linked to ALS and PD, regulates RNA metabolism and mRNA translation, whereas MJD protein ataxin-3 (Atx3) is a deubiquitinase, which trims ubiquitin chains on various target proteins, thereby influencing their stability and function. The calcium voltage-gated channel subunit α1A (CACNA1A) acts as a calcium channel, located in the cell membrane. Besides its causative role in SCA6, non-polyQ mutations in its gene were associated with episodic ataxia type 2 and familial hemiplegic migraine. The SCA7 protein ataxin-7 (Atx7) is a scaffolding component of the multifunctional Spt-Ada-Gcn5 acetyltransferase (SAGA) complex. TATA box-binding protein (TBP) of SCA17 is a general transcription factor, a function shared with the sex hormone-dependent androgen receptor (AR) in SBMA (Orr, 2012; Lieberman et al., 2019; Johnson et al., 2022). PolyQ expansions within these proteins were demonstrated to interfere with their physiological role in a gain-of-function or loss-of-function modality, further explaining the phenotypic distinctness of polyQ disorders.

Despite its relative heterogeneity in symptoms and affected brain areas, and its diversity in the nature of their disease proteins, polyQ disorders also share common features in their pathomechanisms and disease-modifying pathways. These unifying characteristics include the formation of intracellular inclusion bodies - a histological feature of all polyQ diseases - as well as dysregulation in protein clearance mechanisms, nuclear import, gene expression, mitochondrial function, or solute homeostasis, which in consequence trigger neuronal disturbances and eventually cellular demise (Weber et al., 2014; Lieberman et al., 2019; Bunting et al., 2022). Many of these perturbations are modulated by various disease protein-targeting PTMs including phosphorylation, SUMOylation, ubiquitination, as well as proteolytic fragmentation (Matos et al., 2017; Johnson et al., 2022). The latter type of modification represents an important source of deleterious and aggregation-prone fragments, subsumed under the term *toxic fragments*, and calpains - together with caspases - appear to be the key players in the proteolysis of many polyQ proteins. Intriguingly, calpains were reported to be pathologically overactivated in polyQ diseases, a circumstance that might be directly linked to the known disbalance of the calcium signaling homeostasis in neurodegenerative disorders (Bezprozvanny, 2009; Weber et al., 2014; Matos et al., 2017).

#### Calpains in Huntington disease

Proteolytic cleavage as a posttranslational modification of HTT or, more generally, the occurrence of truncated forms of the mutant protein is considered a crucial mediator of polyQ toxicity in the molecular pathogenesis of HD (Wellington and Hayden, 1997; Ehrnhoefer et al., 2011). Different studies demonstrated that N-terminal fragments of HTT containing the polyglutamine expansion were present in the brains of HD patients as well as mouse and cell models (DiFiglia et al., 1997; Tanaka et al., 2006; Schilling et al., 2007; Landles et al., 2010). Importantly, overexpression of such N-terminal HTT fragments in rodent models of HD was sufficient to manifest a progressive disease phenotype (Mangiarini et al., 1996; Davies et al., 1997). Moreover, truncated forms of HTT were reported to translocate into the nucleus, accumulate and form intranuclear aggregates, which eventually induce apoptotic stress and cell death (Hackam et al., 1998; Martindale et al., 1998; Zhou et al., 2003; Landles et al., 2010). Intriguingly, also HTT fragments lacking the polyQ stretch were found to cause cellular dysregulations, e.g., endoplasmic reticulum stress and autophagic perturbations (Martin et al., 2014; El-Daher et al., 2015).

Early reports indicated that cleavage by caspases, especially caspase-6, was a relevant molecular modifier of HD pathogenesis, and inhibiting HTT caspase-dependent cleavage ameliorated multiple disease hallmarks in cell and animal models (Wellington et al., 1998, 2000, 2002; Graham et al., 2006). An alternative non-proteolytic source for truncated HTT was found in a mis-splicing event, which was shown to release a short, toxic, and aggregation-prone exon 1 fragment of the polyQ-expanded protein (Sathasivam et al., 2013).

Just after demonstrating that caspases are involved in HD pathogenesis, researchers focused on evaluating the potential contribution of calpains to the disease pathways. In primary studies, wild-type, as well as mutant HTT, were shown to be substrates of calpain-dependent proteolysis (Gafni and Ellerby, 2002; Goffredo et al., 2002; Gafni et al., 2004). Moreover, caspase cleavage-derived N-terminal fragments of HTT appeared to undergo further calpain-mediated proteolysis (Kim et al., 2001). Importantly, analysis of post-mortem brains of HD patients and mouse models detected elevated levels of calpain-1, -5, -7, and -10. Along with a strong calpain activation, an increased and altered fragmentation pattern of polyQ-expanded HTT was observed in the brains of HD patients (Gafni and Ellerby, 2002; Gafni et al., 2004). Moreover, in an RNA-silencing-based approach, calpain-10 - together with other proteases - was found specifically accountable for the formation of two short N-terminal HTT fragments (Ratovitski et al., 2011). Calpain overactivation at baseline was detected in various HD cell and rodent models, when investigating known calpain substrate proteins such as α-spectrin and p35 (Gafni et al., 2004; Cowan et al., 2008; Paoletti et al., 2008; Clemens et al., 2015; Weber et al., 2016, 2018). This overactivation was also associated with a compromised N-methyl-D-aspartate (NMDA) receptor signaling and excitotoxic effects in HD mice (Cowan et al., 2008; Gladding et al., 2012, 2014).

**Figure 2:**
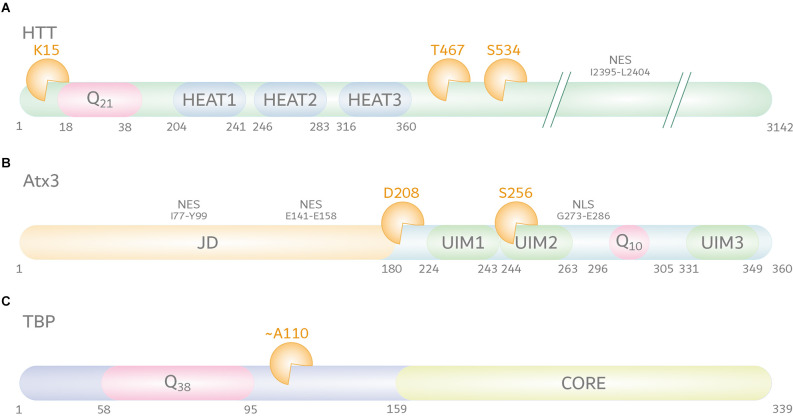
Calpain cleavage sites in huntingtin protein (HTT), ataxin-3 (Atx3), and TATA box-binding protein (TBP). **(A)** HTT (UniProt-ID: P42858) features, aside from its N-terminal polyQ tract, five HEAT repeats, three of the latter being localized within the first 500 amino acids. Moreover, HTT contains a C-terminal nuclear export signal (NES) between amino acid positions I2395-L2404. The best characterized calpain cleavage sites are at amino acids K15, T467, and S534. **(B)** Atx3 (UniProt-ID: P54252-2) consists of an N-terminal proteolytic Josephin domain (JD) carrying two nuclear export signals (NES) between amino acid positions I77-Y99 and E141–E158, and two to three ubiquitin interaction motifs (UIMs). Two UIMs are located N-terminally of the polyQ stretch, while the last isoform-dependent UIM is located at the C-terminus of the protein. A nuclear localization signal (NLS) between amino acid positions G273-E286 is located between UIM2 and the polyQ stretch. The best characterized calpain cleavage sites were mapped to amino acids D208 and S256. **(C)** The location of calpain cleavage sites in TBP (UniProt-ID: P20226-1) is predicted to be C-terminal to its polyQ tract and N-terminal to its core DNA-binding domain, around amino acid position A110. However, the exact cleavage site remains unclear.

Mutating identified calpain cleavage sites at amino acid positions T467 and S534 (positions based on UniProt reference isoform; identifier: P42858; Figure 2A) protected polyQ-expanded HTT from calpain-mediated fragmentation, consequently lowering levels of N-terminal fragments, and reducing HTT aggregation and cytotoxicity (Gafni et al., 2004). Interestingly, mimicking the phosphorylation of S534 by substitution to aspartic acid could strongly lower the cleavage of HTT and reduce the polyQ-mediated toxicity, highlighting the vital crosstalk between different PTMs (Schilling et al., 2006).

Based on the observed calpain overactivation in HD, many studies focused on the potentially protective effects of targeting calpain activity as a therapeutic approach. Overexpression of CAST lowered calpain activation, calpain-dependent HTT fragmentation and aggregation, whereas CAST depletion was associated with calpain overactivation as well as enhanced calpain-mediated HTT cleavage and aggregation *in cellulo* (Weber et al., 2018). Furthermore, in HD knock-in mice, genetic CAST ablation worsened molecular and neuropathological features by further triggering calpain activity, inducing polyQ-expanded HTT cleavage, and increasing fragmentation of additional neuronal substrate proteins (Weber et al., 2018). In line with these findings, calpain knockdown and CAST overexpression *in vivo* showed beneficial effects on mutant HTT aggregation, polyQ toxicity, and HD-related behavior in HTT fragment models, where fragmentation of the disease protein played a subordinate role. These beneficial effects were explained by a calpain inhibition-dependent stimulation of autophagic pathways (Menzies et al., 2015). Interestingly, olesoxime, a neuroprotective cholesterol-like drug candidate which binds the voltage-dependent anion channels (VDACs) and the translocator protein (TSPO) on the outer mitochondrial membrane, was reported to ameliorate disease-related abnormalities in HD rodent models by lowering calpain activity (Clemens et al., 2015; Weber et al., 2016). In these studies, treatment with olesoxime attenuated calpain overactivation, which was accompanied by reduced fragmentation, aggregation, and nuclear accumulation of polyQ-expanded HTT. Importantly, olesoxime administration led to an improvement of the cognitive and psychiatric phenotype as well as reduced brain atrophy in the HD rat model (Clemens et al., 2015). Although the precise mode of action of the molecule remains unknown, it was hypothesized that olesoxime might act beneficially on the mitochondria-endoplasmic calcium coupling in neurons (Weber et al., 2019b). Recently, another small-molecule compound termed CHIR99021, a known glycogen synthase kinase 3 (GSK3) inhibitor, was demonstrated to inhibit calpain overactivation by suppressing proteasomal degradation of CAST in cell and animal models of HD. Notably, CHIR99021 treatment reduced various mitochondrial, neuropathological, and disease-associated hallmarks in HD knock-in mice (Hu et al., 2021). However, this study omitted to investigate the consequences of CHIR99021 administration on HTT fragmentation.

On the other side, calpain-mediated cleavage was also reported to have positive effects on the turnover rate of mutant HTT. In an HD cell model, N-terminal HTT fragments of a calpain-independent origin were shown to be further degraded by calpains, and calpain inhibition led to an accumulation of these breakdown products, accompanied by elevated mutant HTT aggregate formation (Ratovitski et al., 2007). Furthermore, Happ1, an intrabody that binds the proline-rich region of the N-terminus of HTT was shown to enhance the degradation of an exon 1 construct of poly Q-expanded HTT in a calpain-dependent fashion and involving cleavage at K15 (Figure 2A). Reciprocally, another intrabody, which binds to this calpain cleavage site, prevented clearance of the mutant HTT constructs (Southwell et al., 2011).

Aside from its directly linked effects on HTT cleavage and aggregation, treatment with direct or indirect inhibitors of calpain activity or overexpression of CAST proved effective for ameliorating further pathological hallmarks in a multitude of HD cell and animal models. For instance, the administration of calpain inhibitor I/ALLN to medium-sized spiny neurons (MSNs) isolated from HD mice balanced the loss rate of surface NMDA receptors and reduced NMDA-induced apoptosis (Cowan et al., 2008). CAST overexpression as well as CX295 treatments rescued NF-κB-p65 levels in HD cells, thereby lowering oxidative stress and cell degeneration (Reijonen et al., 2010). Likewise, administration of PRE084, a sigma-1 receptor agonist, elevated CAST levels and exerted neuroprotective effects *via* NF-κB-p65 signaling in the same cell model (Hyrskyluoto et al., 2013).

#### Calpains in Machado-Joseph disease

Concurrent with the research history of HD, studies investigated the role of proteolytic cleavage and the validity of the *toxic fragment hypothesis* in the pathological context of MJD. Similar to findings in HD models, early findings in MJD saw correlations between the expression of truncated, polyQ stretch-containing forms of the disease protein Atx3 and increased toxicity, which was accompanied by nuclear mislocalization and aggregation *in vitro* and *in vivo* (Ikeda et al., 1996; Paulson et al., 1997; Goti et al., 2004; Haacke et al., 2006). Fragments of polyQ-expanded Atx3 were detected in the brains of MJD mice and patients and associated with the disease progression (Goti et al., 2004). A subsequent study could narrow down the cleavage site presumably responsible for the observed fragments, but without identifying a responsible protease (Colomer Gould et al., 2007).

Caspases were the first suspects regarding Atx3 cleavage, and some works demonstrated that caspase-dependent fragmentation was indeed occurring *in vitro* and in cell models, however, without detecting reported breakdown products in MJD patient brains (Wellington et al., 1998; Berke et al., 2004). Further supportive data for the involvement of caspases was obtained from a *Drosophila* model of MJD, where caspase cleavage site-resistant Atx3 protected against the polyQ-induced eye degeneration but without effects on disease protein aggregation (Jung et al., 2009).

Primary reports on the involvement of calpains in the fragmentation of polyQ-expanded Atx3 were based on the analysis of proteolytic events observed in neuroblastoma cells. Stimulation of cell lysates with calcium or treatment of cells with a calcium ionophore induced fragmentation of Atx3, which was abolished by the administration of calpain inhibitors, whereas blocking caspases and other proteases did not prevent Atx3 cleavage. CAST overexpression, on the other hand, lowered polyQ-expanded Atx3 cleavage, and aggregation (Haacke et al., 2007). In addition, this study delivered the first information on calpain-specific cleavage sites in the Atx3, which were precisely mapped to two main amino positions at D208 and S256 in a later study (positions based on UniProt reference isoform; identifier: P54252-2; Figure 2B; Haacke et al., 2007; Weber et al., 2017). Fragments derived from calpain cleavage at the identified sites showed strongly increased aggregation propensities and cytotoxicity in cell models of MJD and were found to occur in patient-derived fibroblasts, induced pluripotent stem cells, induced cortical neurons (iCNs), and - most importantly - post-mortem MJD patient brain (Weber et al., 2017). Interestingly, N-terminal fragments of Atx3 lacking the polyQ stretch were shown to induce an MJD-like phenotype in mice and led to mitochondrial perturbations in cell models, pointing toward their participation in the molecular pathogenesis (Hübener et al., 2011; Harmuth et al., 2018).

Based on these findings, several studies targeted the mapped calpain cleavage sites in Atx3 to render the polyQ-expanded protein calpain cleavage-resistant and evaluate the consequences of these modifications on disease hallmarks. For instance, mutating three amino acids around the cleavage sites D208 and S256 to tryptophan residues efficiently abolished cleavage by calpains (Weber et al., 2017). In an antisense oligonucleotide-based exon skipping approach in MJD patient-derived fibroblasts, the removal of two exons of Atx3 ablated the main recognition sites for calpains and caspases of Atx3 and blocked the formation of potentially toxic polyQ-containing fragments. However, due to the exon removal-induced loss of two functionally important ubiquitin interacting motifs in Atx3, as well as low exon skipping efficiencies, this strategy was deemed non-viable as a therapeutic approach (Toonen et al., 2016). In a lentiviral MJD mouse model, deletion of larger amino acid stretches on adjacent calpain cleavage sites within Atx3 reduced disease protein cleavage and aggregation, and retained its cytoplasmic localization (Simões et al., 2022).

Consistent with findings in HD, calpains were not only responsible for the fragmentation of the disease protein but exhibited also an overactivation in respective disease models. A direct link between neuronal specificity of MJD and calpain-mediated cleavage was gained from analyzing the effects of neurotransmitter-induced excitation of MJD patient-derived iCNs. Here, treatment with L-glutamate led to an excitation-dependent calcium influx and, thereby, calpain-dependent cleavage and aggregation of polyQ-expanded Atx3 in iCNs (Koch et al., 2011). Further analysis of MJD patient-derived fibroblasts and MJD animal models delivered additional proof of a pathological calpain overactivation, which caused subsequent proteolytic perturbations in neuronal substrate proteins and might be linked to a described dysregulation of calcium homeostasis by polyQ-expanded Atx3 (Chen et al., 2008; Simões et al., 2012; Weber et al., 2020). Moreover, triggering calpain activation in MJD mice by genetically depleting CAST led to a worsening of the disease-associated molecular and behavioral characteristics, whereas CAST overexpression in a further study ameliorated pathological hallmarks including polyQ-expanded Atx3 cleavage, mislocalization, and aggregation, and neuronal loss (Simões et al., 2012; Hübener et al., 2013). Interestingly, MJD mice harboring a knockout of calpain-1 showed a partially improved phenotype regarding reduced Atx3 cleavage, lowered fragmentation of synaptic proteins, as well as increased body weight and survival, but featured worsened motor symptoms (Weber et al., 2020). These mixed consequences might be explained by the vital role of calpains in neuroprotection and neuronal plasticity and suggest calpain-2 as a more suitable target for therapeutic intervention (Baudry and Bi, 2016).

Aside from genetic strategies targeting cleavage sites in Atx3 or the cleavage-executing calpain system, different studies focused on more clinically translatable approaches using calpain inhibitors. Treatment of MJD patient-derived iCNs with ALLN or calpeptin after excitotoxic L-glutamate stimulation reduced polyQ-expanded Atx3, while caspase-specific inhibitors failed to do so (Koch et al., 2011). Administration of BDA-410, an inhibitor with a relatively higher selectivity for calpain-1 over calpain-2, lowered polyQ-expanded Atx3 cleavage and aggregation, and alleviated neuronal loss and motor symptoms in MJD mice (Li et al., 2007; Simões et al., 2014). Interestingly, two treatment studies using calpain inhibitor calpeptin and BLD-2736, a novel inhibitor of calpain-1, -2, and -9, in MJD zebrafish, did not primarily link the observed beneficial effects on Atx3 aggregation and motor phenotype with a reduced fragmentation of Atx3, but with its higher turnover via the autophagic system (Watchon et al., 2017; Robinson et al., 2021), which is known to be modulated by calpain activity (Weber et al., 2019a).

#### Calpains in spinocerebellar ataxia type 17 and other polyglutamine disorders

The presence of fragments or protease-dependent cleavage of disease proteins was shown to modulate the pathogenesis of SCA1, SCA2, SCA6, SCA7, DRPLA, and SBMA (Weber et al., 2014; Matos et al., 2017). Some studies demonstrated connections to disease protein fragmentation by caspases, e.g., the role of caspase-7-mediated cleavage of Atx7 in SCA7 (Wellington et al., 1998; Young et al., 2007). However, unlike for HD and MJD, the importance of calpains and calpain-mediated disease protein cleavage has been investigated to a lesser extent in the remaining polyQ diseases, leaving many questions - in this context - unanswered.

In the case of SCA17, earlier attempts to associate caspases with the cleavage of the disease protein TBP were inconclusive, despite unequivocal reports on the involvement of truncated forms of polyQ-expanded TBP in the molecular pathology of SCA17 (Wellington et al., 1998; Friedman et al., 2008). In a recent study, new light was shed on TBP fragmentation, detecting calpains as players in the pathogenesis of SCA17 (Weber et al., 2022). In SCA17 cell and rat models, TBP was processed by the overactivated calpain system into prominent C-terminal fragments. Interestingly, in contrast to TBP’s nuclear presence, these arising C-terminal breakdown products were mislocalized to the cytoplasm, suggesting potential negative repercussions on the protein function as a general transcription factor. Importantly, inhibition of calpains by overexpression of CAST or calpain inhibitor administration in SCA17 cells reduced TBP fragmentation, decreased its aggregation, and rescued cell viability impairments, indicating the toxic potential of TBP fragments (Weber et al., 2022). Despite these first significant findings, various aspects of the involvement of calpains in SCA17 remained elusive. Although TBP was suggested to be proteolyzed by calpains C-terminally of the polyQ stretch around amino acid position A110, the exact cleavage site is unclarified (position based on UniProt reference isoform; identifier: P20226-1; Figure 2C; Weber et al., 2022). Moreover, the exact pathological role of calpain cleavage-derived TBP fragments and potential therapeutic interventions on fragmentation and calpain activation by genetic or pharmacologic approaches *in vivo* still have to be examined.

The SCA6 disease protein CACNA1A was found to be cleaved by an unknown protease into a C-terminal fragment, which exhibited increased toxicity and resistance to further proteolysis in cell models (Kubodera et al., 2003). Moreover, this fragment was detected in the post-mortem brains of SCA6 patients and associated with the cytoplasmic aggregation of the disease protein (Ishiguro et al., 2010). Another study, however, showed that shorter, presumably calpain cleavage-independent C-terminal CACNA1A fragments translocate into the nucleus and, thereby, mediate polyglutamine-dependent cytotoxicity (Kordasiewicz et al., 2006). On the other hand, one must consider that any pathological alterations of CACNA1A’s function may affect the cellular calcium homeostasis and consequently induce calpain activation.

Fragments of atrophin-1 (ATN1), the disease protein of DRPLA, were found in cell models, as well as mouse and patient brain, and associated with its polyQ-dependent toxicity. Caspases appeared as primary executors of the underlying proteolytic origin (Miyashita et al., 1997; Ellerby et al., 1999; Schilling et al., 1999). However, two other studies showed that pathologically relevant fragments of ATN1 can also arise in a caspase-independent process (Nucifora et al., 2003; Suzuki et al., 2010). Thus, and despite some opposing indications, calpains cannot be ruled out as potentially responsible proteases in the fragmentation of ATN1 in DRPLA.

While there is evidence for implications of truncated forms of the androgen receptor (AR) in the molecular pathogenesis of SBMA, and caspases have been reported to cleave the disease protein, information on participation of calpains in the disease is scarce (Butler et al., 1998; Kobayashi et al., 1998; Merry et al., 1998; Wellington et al., 1998). However, in an SBMA-independent context, calpain-mediated cleavage of AR was shown to occur in prostate cancer cells rendering the receptor androgen-independent or leading to its elimination (Libertini et al., 2007; Yang et al., 2008). Thus, an involvement of calpains in the cleavage of polyQ-expanded AR is very likely and demands further scrutiny.

Whether disease protein fragmentation must have negative consequences is still debated, with studies challenging - or at least softening - this concept. Contradictory results were found for the SCA2 disease protein Atx2. Here, truncated forms of Atx2 containing the polyglutamine stretch showed high aggregation propensity, and their polyQ flanking regions were crucial for the aggregation process (Nozaki et al., 2001). However, in another study, N-terminally truncated Atx2 fragments did not form aggregates and were less cytotoxic than the full-length protein (Ng et al., 2007). Still, responsible proteases for this process remain to be discovered.

## Conclusion

**Figure 3:**
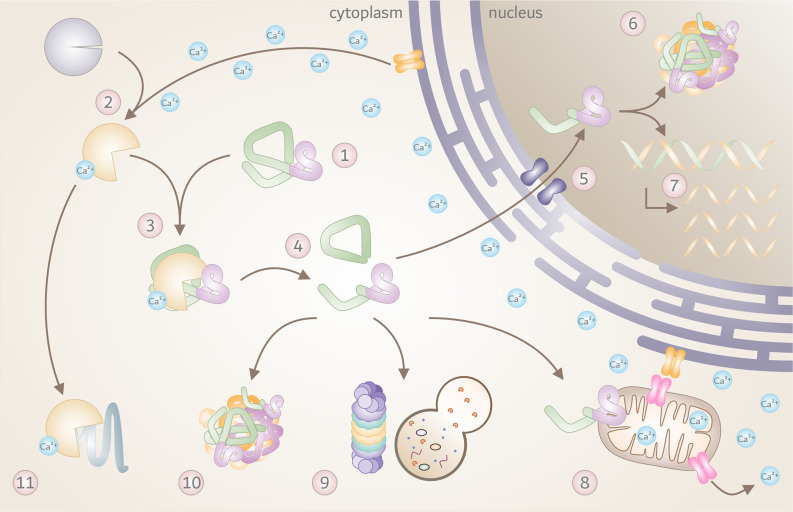
The toxic fragments hypothesis in the molecular pathogenesis of polyQ disorders. Overview of known molecular and cellular repercussions in the context of the pathomechanism of Huntington disease (HD), Machado-Joseph disease (MJD), and spinocerebellar ataxia type 17 (SCA17), with a potential (yet unconfirmed) general validity for all polyQ disorders and beyond. **(1)** PolyQ disorders are caused by mutations that result in a pathologically elongated polyQ tract (highlighted in pink) in the respective disease protein, such as HTT, Atx3 or TBP. **(2)** Due to a pathology-related dysregulation of the cellular calcium homeostasis, calpains become overactivated, and **(3)** engage in cleaving mutant proteins. **(4)** This process results in the formation of toxic disease protein fragments containing the polyQ stretch. **(5)** Breakdown products can translocate to the cell nucleus, **(6)** and accumulate, forming intranuclear inclusions which sequester important nuclear components. **(7)** This process may interfere with transcription, leading to disturbed gene expression. In addition, disease protein fragments can cause **(8)** mitochondrial dysfunction and stress, leading to further calcium disbalance and calpain activation. Disease protein fragments can, furthermore, **(9)** impair protein clearance mechanisms, including autophagy and proteasomal degradation, **(10)** triggering the formation of cytoplasmic aggregates and inclusion bodies. **(11)** Finally, pathologically overactivated calpains may contribute to excessive cleavage of other cellular proteins with negative effects on cell function and viability.

Over the past three decades, a great number of studies corroborated proteolytic fragmentation of disease proteins as highly relevant PTMs, with significant consequences on the molecular mechanisms of neurodegeneration. Based on the findings in HD, MJD, and SCA17 described in this review, the contribution of calpains as the executor of disease protein cleavage within the general mechanisms of polyQ disorders is highly conceivable. A large variety of molecular repercussions may be triggered by the overactivation of calpains and calpain-mediated fragmentation of disease proteins, with detrimental consequences for affected cells as summarized in Figure 3. However, as knowledge of the contribution of calpains in other polyQ diseases - namely SCA1, SCA2, SCA6, SCA7, DRPLA, and SMBA - is fragmentary, additional research efforts are demanded to fully confirm calpain cleavage as a unifying mechanism.

A first step and fast way to gauge whether yet experimentally unverified polyQ proteins might represent calpain substrates is utilizing well-established computational approaches. Tools such as Calpacchopper or GPS-CCD can offer a first insight into the potential proteolytic likelihood and give further information on expected fragment sizes and their composition (DuVerle et al., 2011; Liu et al., 2011; duVerle and Mamitsuka, 2019). The potential involvement of calpains and their pathological activation in underreported polyQ disorders appear, furthermore, very likely, as the intracellular calcium homeostasis - a pivotal factor of their functionality - was shown to be dysregulated in many SCAs (Bezprozvanny, 2009; Kasumu et al., 2012).

A multitude of pharmacologic treatment approaches for lowering detrimental calpain activation have been evaluated in preclinical studies *in vitro* and *in vivo*, with pathology-ameliorating outcomes for polyQ disorders. Novel inhibitors such as BLD-2736, which work at sub-micromolar ranges, showed promising effects in MJD zebrafish (Robinson et al., 2021), and should be further reassessed in additional *in vivo* models, and also for other diseases such as HD and SCA17. A summary of so far tested genetic and direct or indirect pharmacologic strategies targeting the calpain system can be found in Table 2. The overall findings are encouraging. However, the lack of specificity and efficacy of available calpain inhibitors poses a therapeutic obstacle, despite compounds like ABT-957 and BDL-2660 reaching clinical trials (ClinicalTrials.gov, NCT02220738 and NCT04334460) for treatment of AD and COVID-19. In this context, it should not be forgotten that the activity of calpains serves a physiological purpose in a functional biological system. Thus, a misdirected therapeutic interference with this system may lead to unwanted negative repercussions. This circumstance is highlighted by mutations in the human *CAPN1* gene, which reportedly cause spastic paraplegia 76 (SPG76) or a form of cerebellar ataxia (Gan-Or et al., 2016; Wang et al., 2016). Therefore, the development and availability of highly specific inhibitors which only target the desired calpain molecule are essential, as suggested for calpain-2 in the treatment of acute neuronal injury and beyond (Wang et al., 2018). Alternatively, indirect strategies targeting dysfunctional calcium homeostasis, impaired mitochondria, or depletion of CAST levels are auspicious, as demonstrated for compounds like olesoxime and CHIR99021 in HD (Clemens et al., 2015; Weber et al., 2016; Hu et al., 2021). These drugs do not only antagonize overactivated calpains and ameliorate subsequent detrimental effects but also mitigate their underlying disturbances caused by polyQ toxicity. A future translation of these findings to other polyQ disorders and potential clinical trials is necessary to assess their general applicability. Modifying calpains and calpain-mediated cleavage with genetic means has been tested in different experimental set-ups *in vitro* and *in vivo* for HD and SCA3, such as by knocking out specific calpain isoforms (Menzies et al., 2015; Weber et al., 2020), overexpressing the endogenous inhibitor CAST (Simões et al., 2012; Menzies et al., 2015) or removing calpain cleavage sites (Gafni et al., 2004; Toonen et al., 2016; Weber et al., 2017; Simões et al., 2022). Although these strategies led to a therapeutic proof of principle and - in most cases - positive repercussions on certain pathological parameters, the translation into clinical approaches appears intricate, as targeting the mutant disease gene still represents the most direct way.

**Table 2:** Overview of preclinical studies targeting the calpain system in polyQ diseases.

**Compound/Treatment**	**Model**	**Application**	**Outcome**
**Huntington disease**			
*CalpA* RNAi^g,d^ Menzies et al. (2015)	*Drosophila* overexpressing 46Q or 120Q HTT exon 1 fragment	crossbreeding with *CalpA* RNAi fly lines/ 3, 15, 35 dpe	• reduced HTT aggregation • rescue of neurodegeneration in fly eye
CAST^g,d^ Menzies et al. (2015)	N171-82Q mice overexpressing HTT exon 1 fragment	crossbreeding with transgenic mice overexpressing CAST/ up to 19 months of age	• reduced mutant HTT • reduced HTT aggregation • delayed onset of tremors • improved motor function • enhanced locomotor activity • extended life span
CAST ^g,d^ Weber et al. (2018)	HEK 293T cells overexpressing 128Q full-length HTT	vector-based overexpression/ for 72 h	• reduced calpain activity • reduced HTT cleavage • reduced HTT aggregation
CI I/ALLN^p,d^ Cowan et al. (2008)	MSNs derived from YAC72 or YAC128 mice overexpressing 72Q or 128Q full-length human HTT	1 μM/ for 24 h	• normalized loss rate of surface NMDARs to wild-type level • reduced cell death of MSNs treated with NMDA
CI III/MDL 28170^p,d^ Weber et al. (2018)	HEK 293T cells overexpressing 128Q full-length HTT	10 μM/ for 2 h	• reduced calpain activity • reduced HTT cleavage
CX295^p,d^ Reijonen et al. (2010)	PC6.3 cells overexpressing EGFP-HTT exon 1 39/53/120Q or full-length HTT 75Q	20 μM/ for 48 h	• restored NF-κB-p65 levels • restored antioxidant levels • reduced oxidative stress • decreased cell degeneration
CHIR99021^p,i^ Hu et al. (2021)	*Hdh*^Q111^ knock-in cells expressing 111Q full-length HTT	3 μM/ for 48 h	• enhanced mitochondrial function • enhanced cell viability • reduced calpain activity • restored CAST levels
	HD patient-derived neuronal cells and fibroblasts	1 μM/ for 96–120 h	
	R6/2 mice overexpressing 100-150Q N-terminal HTT fragment	i.p. injection, 10 μg/g/ 5 days/week starting from 6 weeks until 12 weeks of age	• reduced calpain activity • restored CAST levels • reduced HTT aggregation • attenuated motor deficits • enhanced locomotor activity improved survival	
	YAC128 mice overexpressing 128Q full-length human HTT	i.p. injection, 10 μg/g/ once every other day starting from 9 months until 12 months of age	
Olesoxime^p,i^ Clemens et al. (2015)	BACHD rats overexpressing 97Q full-length HTT	orally *via* food, *ad libitum*, 600 μg/g/ for 12 months starting at 5 weeks of age	• reduced calpain activity • reduced HTT fragments • reduced aggregation and nuclear accumulation of mutant HTT • improved mitochondrial function • amelioration of brain atrophy • improvement of cognitive and psychiatric signs
Olesoxime^p,i^ Weber et al. (2016)	*Hdh*^Q111^ knock-in mice expressing 111Q full-length HTT	orally *via* food, *ad libitum*, 600 μg/g/ for 3 months starting prenatally	• reduced calpain activity • reduced HTT fragments
PRE084^p,i^ Hyrskyluoto et al. (2013)	PC6.3 cells overexpressing EGFP-HTT exon 1 120Q or full-length HTT 75Q	300 nM PRE084/ for 24 h, 48 h	• restored CAST levels • restored NF-κB-p65 levels and signaling • neuroprotective effects
**Machado-Joseph disease**			
BDA-410^p,d^ Simões et al. (2014)	LV mouse model overexpressing 72Q full-length human Atx3	daily oral gavage, 30 μg/g/ for 4–13 weeks	• reduced Atx3 cleavage • reduced Atx3 aggregation • reduced cytotoxicity • alleviated neurodegeneration • improved motor coordination
BLD-2736^p,d^ Robinson et al. (2021)	MJD zebrafish overexpressing Atx3 84Q in neurons	150, 225, and 300 nM/ for 5 days (1 dpf - 6 dpf) or for 2 days (4 dpf - 6 dpf)	• reduced Atx3 cleavage • reduced levels of soluble and aggregated Atx3 • increased autophagic flux • improved motor phenotype
Calpeptin^p,d^ Haacke et al. (2007)	N2a cells expressing Atx3 71Q	5, 50, 200 μM/ for 2 h or 50 μM/for 72 h	• reduced Atx3 cleavage upon ionomycin-treatment • reduced Atx3 aggregation
Calpeptin^p,d^ Koch et al. (2011)	MJD patient-derived iCNs	20, 200 μM/ for 24 h	• reduced Atx3 aggregation after L-glutamate-induced excitation
Calpeptin^p,d^ Watchon et al. (2017) and Robinson et al. (2021)	MJD zebrafish overexpressing Atx3 84Q in neurons	6.25–100 μM/ for 2 days (1 dpf -2 dpf) or 5 days (1 dpf -6 dpf)	• reduced Atx3 cleavage • reduced levels of soluble and aggregated Atx3 • increased autophagic flux • improved motor phenotype
*CAPN1* KD^g,d^ or *Capn1* KO^g,d^ Weber et al. (2020)	HEK 239T cells expressing 70Q full-length human Atx3	esiRNA-mediated knockdown/ for 48 h	• reduced calpain activity • reduced Atx3 fragmentation
	YAQ84Q mice overexpressing human full-length Atx3	crossbreeding with *Capn1* KOmice/ up to 18 months of age	• reduced calpain activity • reduced Atx3 fragmentation • reduced cleavage of synaptic proteins • improved body weight • extended life span
CAST^g,d^ Haacke et al. (2007)	HEK 293T cells expressing Atx3 71Q	vector-based overexpression/ for 24–72 h	• reduced Atx3 cleavage upon ionomycin-treatment • reduced Atx3 aggregation
CAST^g,d^ Simões et al. (2012)	LV mouse model overexpressing 72Q full-length human Atx3	single injection into striatum of AAV vectors encoding calpastatin	• reduced Atx3 proteolysis • reduced Atx3 aggregation • prevention of nuclear translocation of Atx3 • alleviated neurodegeneration
CI I/ALLN^p,d^ Koch et al. (2011)	MJD patient-derived iCNs	10, 100 μM/ for 24 h	• reduced Atx3 aggregation after L-glutamate-induced excitation
CI III/MDL 28170^p,d^ Weber et al. (2017)	MJD patient-derived fibroblasts	10 μM/ for 2 h	• reduced calpain activity • reduced Atx3 fragmentation
**Spinocerebellar ataxia type 17**			
CAST^g,d^ Weber et al. (2022)	HEK 293T cells overexpressing 64Q/105Q full-length TBP	vector-based overexpression/ for 72 h	• reduced calpain activation • reduced TBP cleavage • reduced TBP aggregation • improved cell viability
CI III/MDL 28170^p,d^ Weber et al. (2022)	HEK 293T and PC12 cells overexpressing 64Q/105Q full-length TBP	25 μM/ for 3 h–48 h	• reduced calpain activation • reduced TBP cleavage • reduced TBP aggregation

Summary of cell-based and *in vivo* studies, grouped by polyQ disease, and sorted alphabetically. Experimental specifications and study outcomes are listed. The table comprises both genetic and pharmacologic approaches, acting directly or indirectly on the calpain system. AAV, adeno-associated virus; *CalpA*, *Drosophila* calpain-A gene; *Capn1*, mouse calpain-1 gene; *CAPN1*, human calpain-1 gene; CI I, calpain inhibitor I; CI III, calpain inhibitor III; dpe, days post eclosion; dpf, days post fertilization; iCNs, induced cortical neurons; i.p., intraperitoneal; KD, knockdown; KO, knockout; LV, lentiviral; MSNs, medium-sized spiny neurons; NMDA(R), N-methyl-D-aspartate (receptor); RNAi, RNA interference; ^d^direct inhibition; ^g^genetic; ^i^indirect inhibition; ^p^pharmacologic.

The biological function of the wild-type polyQ protein cleavage by calpains, which act in a modulatory way, remains largely unclarified. Both HTT and Atx3 undergo calpain-mediated proteolysis at baseline regardless of their polyQ-expansion, suggesting a natural function behind this process (Weber et al., 2017, 2018). Investigating the consequences of calpain-mediated cleavage on the protein’s and its fragments’ function, intracellular localization, interactome, and stability might generate a better understanding of both physiological and pathological mechanisms in the respective disease context.

Only by broadening our perspective and deepening our insights into the role of posttranslational proteolytic events mediated by calpains - not only in polyQ disorders but also in other neurodegenerative diseases - will allow us to understand and harness the full therapeutic potential of calpains.

## Author Contributions

RI, HN, and JW conceptualized the manuscript. RI and JW wrote the initial version of the manuscript. RI created the illustrations. All authors contributed to the article and approved the submitted version.

## Funding

HN and JW received funding from the German Research Foundation (Deutsche Forschungsgemeinschaft, DFG; research grant numbers NG 101/6-1 | WE 6585/1-1). We acknowledge support by the Open Access Publication Funds of the Ruhr-Universität Bochum.

## Conflict of Interest

The authors declare that the research was conducted in the absence of any commercial or financial relationships that could be construed as a potential conflict of interest.

## Publisher’s Note

All claims expressed in this article are solely those of the authors and do not necessarily represent those of their affiliated organizations, or those of the publisher, the editors and the reviewers. Any product that may be evaluated in this article, or claim that may be made by its manufacturer, is not guaranteed or endorsed by the publisher.

## References

[B1] Arthur J. S. Elce J. S. Hegadorn C. Williams K. Greer P. A. (2000). Disruption of the murine calpain small subunit gene, Capn4: calpain is essential for embryonic development but not for cell growth and division. Mol. Cell Biol. 20, 4474–4481. 10.1128/MCB.20.12.4474-4481.2000 10825211 PMC85815

[B2] Arthur J. S. Gauthier S. Elce J. S. (1995). Active site residues in m-calpain: identification by site-directed mutagenesis. FEBS Lett. 368, 397–400. 10.1016/0014-5793(95)00691-2 7635186

[B3] Baudry M. Bi X. (2016). Calpain-1 and Calpain-2: the Yin and Yang of synaptic plasticity and neurodegeneration. Trends Neurosci. 39, 235–245. 10.1016/j.tins.2016.01.007 26874794 PMC4818674

[B4] Baudry M. Su W. Seinfeld J. Sun J. Bi X. (2021). Role of Calpain-1 in neurogenesis. Front. Mol. Biosci. 8:685938. 10.3389/fmolb.2021.685938 34212005 PMC8239220

[B5] Berke S. J. S. Schmied F. a. F. Brunt E. R. Ellerby L. M. Paulson H. L. (2004). Caspase-mediated proteolysis of the polyglutamine disease protein ataxin-3. J. Neurochem. 89, 908–918. 10.1111/j.1471-4159.2004.02369.x 15140190

[B6] Bezprozvanny I. (2009). Calcium signaling and neurodegenerative diseases. Trends Mol. Med. 15, 89–100. 10.1016/j.molmed.2009.01.001 19230774 PMC3226745

[B7] Blanchard H. Grochulski P. Li Y. Arthur J. S. C. Davies P. L. Elce J. S.. (1997). Structure of a calpain Ca^2+^-binding domain reveals a novel EF-hand Ca2+-induced conformational changes. Nat. Struct. Biol. 4, 532–538. 10.1038/nsb0797-532 9228945

[B8] Bluhm A. Schrempel S. von Hörsten S. Schulze A. RoSSner S. (2021). Proteolytic α-Synuclein cleavage in health and disease. Int. J. Mol. Sci. 22:5450. 10.3390/ijms22115450 34064208 PMC8196865

[B9] Bochner R. Samuelov L. Sarig O. Li Q. Adase C. A. Isakov O.. (2017). Calpain 12 function revealed through the study of an atypical case of autosomal recessive congenital ichthyosis. J. Invest. Dermatol. 137, 385–393. 10.1016/j.jid.2016.07.043 27769845

[B10] Bunting E. L. Hamilton J. Tabrizi S. J. (2022). Polyglutamine diseases. Curr. Opin. Neurobiol. 72, 39–47. 10.1016/j.conb.2021.07.001 34488036

[B11] Butler R. Leigh P. N. McPhaul M. J. Gallo J. M. (1998). Truncated forms of the androgen receptor are associated with polyglutamine expansion in X-linked spinal and bulbar muscular atrophy. Hum. Mol. Genet. 7, 121–127. 10.1093/hmg/7.1.121 9384612

[B13] Chen Z. Sequeiros J. Tang B. Jiang H. (2018). Genetic modifiers of age-at-onset in polyglutamine diseases. Ageing Res. Rev. 48, 99–108. 10.1016/j.arr.2018.10.004 30355507

[B12] Chen X. Tang T.-S. T.-S. Tu H. Nelson O. Pook M. Hammer R.. (2008). Deranged calcium signaling and neurodegeneration in spinocerebellar ataxia type 3. J. Neurosci. 28, 12713–12724. 10.1523/JNEUROSCI.3909-08.2008 19036964 PMC2663415

[B14] Chhangani D. Martín-Peña A. Rincon-Limas D. E. (2021). Molecular, functional and pathological aspects of TDP-43 fragmentation. iScience 24:102459. 10.1016/j.isci.2021.102459 34013172 PMC8113996

[B15] Clemens L. E. Weber J. J. Wlodkowski T. T. Yu-Taeger L. Michaud M. Calaminus C.. (2015). Olesoxime suppresses calpain activation and mutant huntingtin fragmentation in the BACHD rat. Brain 138, 3632–3653. 10.1093/brain/awv290 26490331

[B16] Colomer Gould V. F. Goti D. Pearce D. Gonzalez G. A. Gao H. Bermudez de Leon M.. (2007). A mutant ataxin-3 fragment results from processing at a site N-terminal to amino acid 190 in brain of Machado-Joseph disease-like transgenic mice. Neurobiol. Dis. 27, 362–369. 10.1016/j.nbd.2007.06.005 17632007 PMC2040168

[B17] Cowan C. M. Fan M. M. Fan J. Shehadeh J. Zhang L. Y. Graham R. K.. (2008). Polyglutamine-modulated striatal calpain activity in YAC transgenic huntington disease mouse model: impact on NMDA receptor function and toxicity. J. Neurosci. 28, 12725–12735. 10.1523/JNEUROSCI.4619-08.2008 19036965 PMC6671821

[B18] Davies S. W. Turmaine M. Cozens B. a. DiFiglia M. Sharp a H. Ross C. a, et al. (1997). Formation of neuronal intranuclear inclusions underlies the neurological dysfunction in mice transgenic for the HD mutation. Cell 90, 537–548. 10.1016/s0092-8674(00)80513-9 9267033

[B19] de Strooper B. Annaert W. (2000). Proteolytic processing and cell biological functions of the amyloid precursor protein. J. Cell Sci. 113, 1857–1870. 10.1242/jcs.113.11.1857 10806097

[B20] Dear T. N. Meier N. T. Hunn M. Boehm T. (2000). Gene structure, chromosomal localization and expression pattern of Capn12, a new member of the calpain large subunit gene family. Genomics 68, 152–160. 10.1006/geno.2000.6289 10964513

[B21] DiFiglia M. Sapp E. Chase K. O. Davies S. W. Bates G. P. Vonsattel J. P.. (1997). Aggregation of huntingtin in neuronal intranuclear inclusions and dystrophic neurites in brain. Science 277, 1990–1993. 10.1126/science.277.5334.1990 9302293

[B22] duVerle D. A. Mamitsuka H. (2019). CalCleaveMKL: a tool for calpain cleavage prediction. Methods Mol. Biol. 1915, 121–147. 10.1007/978-1-4939-8988-1_11 30617801

[B23] DuVerle D. A. Ono Y. Sorimachi H. Mamitsuka H. (2011). Calpain cleavage prediction using multiple kernel learning. PLoS One 6:e19035. 10.1371/journal.pone.0019035 21559271 PMC3086883

[B24] Ehrnhoefer D. E. Sutton L. Hayden M. R. (2011). Small changes, big impact: posttranslational modifications and function of huntingtin in Huntington disease. Neuroscientist 17, 475–492. 10.1177/1073858410390378 21311053 PMC3200085

[B25] El-Daher M.-T. Hangen E. Bruyère J. Poizat G. Al-Ramahi I. Pardo R.. (2015). Huntingtin proteolysis releases non-polyQ fragments that cause toxicity through dynamin 1 dysregulation. EMBO J. 34, 2255–2271. 10.15252/embj.201490808 26165689 PMC4585462

[B26] Ellerby L. M. Andrusiak R. L. Wellington C. L. Hackam A. S. Propp S. S. Wood J. D.. (1999). Cleavage of atrophin-1 at caspase site aspartic acid 109 modulates cytotoxicity. J. Biol. Chem. 274, 8730–8736. 10.1074/jbc.274.13.8730 10085113

[B27] Esch F. S. Keim P. S. Beattie E. C. Blacher R. W. Culwell A. R. Oltersdorf T.. (1990). Cleavage of amyloid beta peptide during constitutive processing of its precursor. Science 248, 1122–1124. 10.1126/science.2111583 2111583

[B28] Esler W. P. Wolfe M. S. (2001). A portrait of Alzheimer secretases—new features and familiar faces. Science 293, 1449—1454. 10.1126/science.1064638 11520976

[B29] Friedman M. J. Wang C.-E. Li X.-J. Li S. (2008). Polyglutamine expansion reduces the association of TATA-binding protein with DNA and induces DNA binding-independent neurotoxicity. J. Biol. Chem. 283, 8283–8290. 10.1074/jbc.M709674200 18218637 PMC2276379

[B31] Gafni J. Cong X. Chen S. F. Gibson B. W. Ellerby L. M. (2009). Calpain-1 cleaves and activates caspase-7. J. Biol. Chem. 284, 25441–25449. 10.1074/jbc.M109.038174 19617626 PMC2757245

[B30] Gafni J. Ellerby L. M. (2002). Calpain activation in Huntington’s disease. J. Neurosci. 22, 4842–4849. 10.1523/JNEUROSCI.22-12-04842.2002 12077181 PMC6757710

[B32] Gafni J. Hermel E. Young J. E. Wellington C. L. Hayden M. R. Ellerby L. M. (2004). Inhibition of calpain cleavage of huntingtin reduces toxicity: accumulation of calpain/caspase fragments in the nucleus. J. Biol. Chem. 279, 20211–20220. 10.1074/jbc.M401267200 14981075

[B33] Gallardo E. Saenz A. Illa I. (2011). Limb-girdle muscular dystrophy 2A. Handb. Clin. Neurol. 101, 97–110. 10.1016/B978-0-08-045031-5.00006-2 21496626

[B34] Gan-Or Z. Bouslam N. Birouk N. Lissouba A. Chambers D. B. Vérièpe J.. (2016). Mutations in CAPN1 cause autosomal-recessive hereditary spastic paraplegia. Am. J. Hum. Genet. 98, 1038–1046. 10.1016/j.ajhg.2016.04.002 27153400 PMC4863665

[B35] Gladding C. M. Fan J. Zhang L. Y. Wang L. Xu J. Li E. H.. (2014). Alterations in STriatal-Enriched protein tyrosine Phosphatase expression, activation and downstream signaling in early and late stages of the YAC128 Huntington’s disease mouse model. J. Neurochem. 130, 145–159. 10.1111/jnc.12700 24588402 PMC4065618

[B36] Gladding C. M. Sepers M. D. Xu J. Zhang L. Y. Milnerwood A. J. Lombroso P. J.. (2012). Calpain and STriatal-Enriched protein tyrosine phosphatase (STEP) activation contribute to extrasynaptic NMDA receptor localization in a Huntington’s disease mouse model. Hum. Mol. Genet. 21, 3739–3752. 10.1093/hmg/dds154 22523092 PMC3412376

[B37] Goffredo D. Rigamonti D. Tartari M. de Micheli A. Verderio C. Matteoli M.. (2002). Calcium-dependent cleavage of endogenous wild-type huntingtin in primary cortical neurons. J. Biol. Chem. 277, 39594–39598. 10.1074/jbc.C200353200 12200414

[B38] Goll D. E. Thompson V. F. Li H. Wei W. Cong J. (2003). The calpain system. Physiol. Rev. 83, 731–801. 10.1152/physrev.00029.2002 12843408

[B39] Goti D. Katzen S. M. Mez J. Kurtis N. Kiluk J. Ben-Haïem L.. (2004). A Mutant Ataxin-3 putative-cleavage fragment in brains of Machado-Joseph disease patients and transgenic mice is cytotoxic above a critical concentration. J. Neurosci. 24, 10266–10279. 10.1523/JNEUROSCI.2734-04.2004 15537899 PMC6730179

[B40] Graham R. K. Deng Y. Slow E. J. Haigh B. Bissada N. Lu G.. (2006). Cleavage at the caspase-6 site is required for neuronal dysfunction and degeneration due to mutant huntingtin. Cell 125, 1179–1191. 10.1016/j.cell.2006.04.026 16777606

[B41] Guroff G. (1964). A neutral, calcium-activated proteinase from the soluble fraction of rat brain. J. Biol. Chem. 239, 149–155. 14114836

[B42] Haacke A. Broadley S. a. Boteva R. Tzvetkov N. Hartl F. U. Breuer P. (2006). Proteolytic cleavage of polyglutamine-expanded ataxin-3 is critical for aggregation and sequestration of non-expanded ataxin-3. Hum. Mol. Genet. 15, 555–568. 10.1093/hmg/ddi472 16407371

[B43] Haacke A. Hartl F. U. Breuer P. (2007). Calpain inhibition is sufficient to suppress aggregation of polyglutamine-expanded ataxin-3. J. Biol. Chem. 282, 18851–18856. 10.1074/jbc.M611914200 17488727

[B44] Hackam A. S. Singaraja R. Wellington C. L. Metzler M. McCutcheon K. Zhang T.. (1998). The influence of huntingtin protein size on nuclear localization and cellular toxicity. J. Cell Biol. 141, 1097–1105. 10.1083/jcb.141.5.1097 9606203 PMC2137174

[B45] Hanna R. A. Campbell R. L. Davies P. L. (2008). Calcium-bound structure of calpain and its mechanism of inhibition by calpastatin. Nature 456, 409–412. 10.1038/nature07451 19020623

[B46] Harmuth T. Prell-Schicker C. Weber J. J. Gellerich F. Funke C. Drießen S.. (2018). Mitochondrial morphology, function and homeostasis are impaired by expression of an N-terminal calpain cleavage fragment of Ataxin-3. Front. Mol. Neurosci. 11:368. 10.3389/fnmol.2018.00368 30364204 PMC6192284

[B47] Hata S. Abe M. Suzuki H. Kitamura F. Toyama-Sorimachi N. Abe K.. (2010). Calpain 8/nCL-2 and calpain 9/nCL-4 constitute an active protease complex, G-calpain, involved in gastric mucosal defense. PLoS Genet. 6:e1001040. 10.1371/journal.pgen.1001040 20686710 PMC2912385

[B48] Horikawa Y. Oda N. Cox N. J. Li X. Orho-Melander M. Hara M.. (2000). Genetic variation in the gene encoding calpain-10 is associated with type 2 diabetes mellitus. Nat. Genet. 26, 163–175. 10.1038/79876 11017071

[B49] Hu D. Sun X. Magpusao A. Fedorov Y. Thompson M. Wang B.. (2021). Small-molecule suppression of calpastatin degradation reduces neuropathology in models of Huntington’s disease. Nat. Commun. 12:5305. 10.1038/s41467-021-25651-y 34489447 PMC8421361

[B50] Hübener J. Vauti F. Funke C. Wolburg H. Ye Y. Schmidt T.. (2011). N-terminal ataxin-3 causes neurological symptoms with inclusions, endoplasmic reticulum stress and ribosomal dislocation. Brain 134, 1925–1942. 10.1016/j.amj.2022.04.004 21653538

[B51] Hübener J. Weber J. J. Richter C. Honold L. Weiss A. Murad F.. (2013). Calpain-mediated ataxin-3 cleavage in the molecular pathogenesis of spinocerebellar ataxia type 3 (SCA3). Hum. Mol. Genet. 22, 508–518. 23100324 10.1093/hmg/dds449

[B52] Hyrskyluoto A. Pulli I. Törnqvist K. Ho T. H. Korhonen L. Lindholm D. (2013). Sigma-1 receptor agonist PRE084 is protective against mutant huntingtin-induced cell degeneration: involvement of calpastatin and the NF-κB pathway. Cell Death Dis. 4:e646. 10.1038/cddis.2013.170 23703391 PMC3674377

[B53] Ikeda H. Yamaguchi M. Sugai S. Aze Y. Narumiya S. Kakizuka A. (1996). Expanded polyglutamine in the Machado-Joseph disease protein induces cell death in vitro and in vivo. Nat. Genet. 13, 196–202. 10.1038/ng0696-196 8640226

[B54] Ishiguro T. Ishikawa K. Takahashi M. Obayashi M. Amino T. Sato N.. (2010). The carboxy-terminal fragment of alpha(1A) calcium channel preferentially aggregates in the cytoplasm of human spinocerebellar ataxia type 6 Purkinje cells. Acta Neuropathol. 119, 447–464. 10.1007/s00401-009-0630-0 20043227 PMC2841749

[B55] Johnson S. L. Tsou W.-L. Prifti M. V. Harris A. L. Todi S. V. (2022). A survey of protein interactions and posttranslational modifications that influence the polyglutamine diseases. Front. Mol. Neurosci. 15:974167. 10.3389/fnmol.2022.974167 PMC9515312 36187346

[B56] Jung J. Xu K. Lessing D. Bonini N. M. (2009). Preventing Ataxin-3 protein cleavage mitigates degeneration in a *Drosophila* model of SCA3. Hum. Mol. Genet. 18, 4843–4852. 10.1093/hmg/ddp456 19783548 PMC2778376

[B57] Kasumu A. W. Liang X. Egorova P. Vorontsova D. Bezprozvanny I. (2012). Chronic suppression of inositol 1,4,5-triphosphate receptor-mediated calcium signaling in cerebellar purkinje cells alleviates pathological phenotype in spinocerebellar ataxia 2 mice. J. Neurosci. 32, 12786–12796. 10.1523/JNEUROSCI.1643-12.2012 22973002 PMC3470884

[B58] Kim Y. J. Yi Y. Sapp E. Wang Y. Cuiffo B. Kegel K. B.. (2001). Caspase 3-cleaved N-terminal fragments of wild-type and mutant huntingtin are present in normal and Huntington’s disease brains, associate with membranes and undergo calpain-dependent proteolysis. Proc. Natl. Acad. Sci. U S A 98, 12784–12789. 10.1073/pnas.221451398 11675509 PMC60131

[B59] Klein T. Eckhard U. Dufour A. Solis N. Overall C. M. (2018). Proteolytic cleavage-mechanisms, function and “Omic” approaches for a near-ubiquitous posttranslational modification. Chem. Rev. 118, 1137–1168. 10.1021/acs.chemrev.7b00120 29265812

[B60] Kobayashi Y. Miwa S. Merry D. E. Kume A. Mei L. Doyu M.. (1998). Caspase-3 cleaves the expanded androgen receptor protein of spinal and bulbar muscular atrophy in a polyglutamine repeat length-dependent manner. Biochem. Biophys. Res. Commun. 252, 145–150. 10.1006/bbrc.1998.9624 9813160

[B61] Koch P. Breuer P. Peitz M. Jungverdorben J. Kesavan J. Poppe D.. (2011). Excitation-induced ataxin-3 aggregation in neurons from patients with Machado-Joseph disease. Nature 480, 543–546. 10.1038/nature10671 22113611

[B62] Kordasiewicz H. B. Thompson R. M. Clark H. B. Gomez C. M. (2006). C-termini of P/Q-type Ca^2+^ channel α1A subunits translocate to nuclei and promote polyglutamine-mediated toxicity. Hum. Mol. Genet. 15, 1587–1599. 10.1093/hmg/ddl080 16595610

[B63] Kottyan L. C. Davis B. P. Sherrill J. D. Liu K. Rochman M. Kaufman K.. (2014). Genome-wide association analysis of eosinophilic esophagitis provides insight into the tissue specificity of this allergic disease. Nat. Genet. 46, 895–900. 10.1038/ng.3033 25017104 PMC4121957

[B64] Kubodera T. Yokota T. Ohwada K. Ishikawa K. Miura H. Matsuoka T.. (2003). Proteolytic cleavage and cellular toxicity of the human α1A calcium channel in spinocerebellar ataxia type 6. Neurosci. Lett. 341, 74–78. 10.1016/s0304-3940(03)00156-3 12676347

[B65] LaFerla F. M. Green K. N. Oddo S. (2007). Intracellular amyloid-β in Alzheimer’s disease. Nat. Rev. Neurosci. 8, 499–509. 10.1038/nrn2168 17551515

[B66] Landles C. Sathasivam K. Weiss A. Woodman B. Moffitt H. Finkbeiner S.. (2010). Proteolysis of mutant huntingtin produces an exon 1 fragment that accumulates as an aggregated protein in neuronal nuclei in Huntington disease. J. Biol. Chem. 285, 8808–8823. 10.1074/jbc.M109.075028 20086007 PMC2838303

[B67] Li X. Chen H. Jeong J. J. Chishti A. H. (2007). BDA-410: a novel synthetic calpain inhibitor active against blood stage malaria. Mol. Biochem. Parasitol. 155, 26–32. 10.1016/j.molbiopara.2007.05.004 17583361 PMC1993804

[B68] Libertini S. J. Tepper C. G. Rodriguez V. Asmuth D. M. Kung H. J. Mudryj M. (2007). Evidence for calpain-mediated androgen receptor cleavage as a mechanism for androgen independence. Cancer Res. 67, 9001–9005. 10.1158/0008-5472.CAN-07-1072 17909000

[B69] Lieberman A. P. Shakkottai V. G. Albin R. L. (2019). Polyglutamine repeats in neurodegenerative diseases. Annu. Rev. Pathol. 14, 1–27. 10.1146/annurev-pathmechdis-012418-012857 30089230 PMC6387631

[B70] Lin G. d. Chattopadhyay D. Maki M. Wang K. K. W. Carson M. Jin L.. (1997). Crystal structure of calcium bound domain VI of calpain at 1.9 A resolution and its role in enzyme assembly, regulation and inhibitor binding. Nat. Struct. Biol. 4, 539–547. 10.1038/nsb0797-539 9228946

[B71] Lin Z. Zhao J. Nitoiu D. Scott C. A. Plagnol V. Smith F. J.. (2015). Loss-of-function mutations in CAST cause peeling skin, leukonychia, acral punctate keratoses, cheilitis and knuckle pads. Am. J. Hum. Genet. 96, 440–447. 10.1016/j.ajhg.2014.12.026 25683118 PMC4375526

[B72] Liu Z. Cao J. Gao X. Ma Q. Ren J. Xue Y. (2011). GPS-CCD: a novel computational program for the prediction of calpain cleavage sites. PLoS One 6:e19001. 10.1371/journal.pone.0019001 21533053 PMC3080405

[B200] Ma H. Nakajima E. Shih M. Azuma M. Shearer T. R. (2004). Expression of calpain small subunit 2 in mammalian tissues. Curr. Eye Res. 29, 337–347. 10.1080/02713680490516242 15590481

[B73] Mahajan V. B. Skeie J. M. Bassuk A. G. Fingert J. H. Braun T. A. Daggett H. T.. (2012). Calpain-5 mutations cause autoimmune uveitis, retinal neovascularization and photoreceptor degeneration. PLoS Genet. 8:e1003001. 10.1371/journal.pgen.1003001 23055945 PMC3464205

[B74] Mangiarini L. Sathasivam K. Seller M. Cozens B. Harper A. Hetherington C.. (1996). Exon 1 of the HD gene with an expanded CAG repeat is sufficient to cause a progressive neurological phenotype in transgenic mice. Cell 87, 493–506. 10.1016/s0092-8674(00)81369-0 8898202

[B75] Martin D. D. O. Heit R. J. Yap M. C. Davidson M. W. Hayden M. R. Berthiaume L. G. (2014). Identification of a post-translationally myristoylated autophagy-inducing domain released by caspase cleavage of huntingtin. Hum. Mol. Genet. 23, 3166–3179. 10.1093/hmg/ddu027 24459296 PMC4030772

[B76] Martindale D. Hackam A. Wieczorek A. Ellerby L. Wellington C. McCutcheon K.. (1998). Length of huntingtin and its polyglutamine tract influences localization and frequency of intracellular aggregates. Nat. Genet. 18, 150–154. 10.1038/ng0298-150 9462744

[B77] Matos C. A. Almeida L. P. d. Nobrega C. (2017). Proteolytic cleavage of polyglutamine disease-causing proteins: revisiting the toxic fragment hypothesis. Curr. Pharm. Des. 23, 753–775. 10.2174/1381612822666161227121912 28025946

[B78] McMurray C. T. (2010). Mechanisms of trinucleotide repeat instability during human development. Nat. Rev. Genet. 11, 786–799. 10.1038/nrg2828 20953213 PMC3175376

[B79] Menzies F. M. Garcia-Arencibia M. Imarisio S. O’Sullivan N. C. Ricketts T. Kent B. a.. (2015). Calpain inhibition mediates autophagy-dependent protection against polyglutamine toxicity. Cell Death Differ. 22, 433–444. 10.1038/cdd.2014.151 25257175 PMC4326573

[B80] Merry D. E. Kobayashi Y. Bailey C. K. Taye A. A. Fischbeck K. H. (1998). Cleavage, aggregation and toxicity of the expanded androgen receptor in spinal and bulbar muscular atrophy. Hum. Mol. Genet. 7, 693–701. 10.1093/hmg/7.4.693 9499423

[B81] Miyashita T. Okamura-Oho Y. Mito Y. Nagafuchi S. Yamada M. (1997). Dentatorubral pallidoluysian atrophy (DRPLA) protein is cleaved by caspase-3 during apoptosis. J. Biol. Chem. 272, 29238–29242. 10.1074/jbc.272.46.29238 9361003

[B82] Moldoveanu T. Gehring K. Green D. R. (2008). Concerted multi-pronged attack by calpastatin to occlude the catalytic cleft of heterodimeric calpains. Nature 456, 404–408. 10.1038/nature07353 19020622 PMC2847431

[B83] Moldoveanu T. Hosfield C. M. Lim D. Elce J. S. Jia Z. Davies P. L. (2002). A Ca^2+^ switch aligns the active site of calpain. Cell 108, 649–660. 10.1016/s0092-8674(02)00659-1 11893336

[B84] Murachi T. Tanaka K. Hatanaka M. Murakami T. (1980). Intracellular Ca^2+^-dependent protease (calpain) and its high-molecular-weight endogenous inhibitor (calpastatin). Adv. Enzyme Regul. 19, 407–424. 10.1016/0065-2571(81)90026-1 6278869

[B85] Ng H. Pulst S.-M. Huynh D. P. (2007). Ataxin-2 mediated cell death is dependent on domains downstream of the polyQ repeat. Exp. Neurol. 208, 207–215. 10.1016/j.expneurol.2007.07.023 17949716 PMC2186298

[B86] Nian H. Ma B. (2021). Calpain-calpastatin system and cancer progression. Biol. Rev. Camb. Philos. Soc. 96, 961–975. 10.1111/brv.12686 33470511

[B87] Nozaki K. Onodera O. Takano H. Tsuji S. (2001). Amino acid sequences flanking polyglutamine stretches influence their potential for aggregate formation. Neuroreport 12, 3357–3364. 10.1097/00001756-200110290-00042 11711886

[B88] Nucifora F. C. Ellerby L. M. Wellington C. L. Wood J. D. Herring W. J. Sawa A.. (2003). Nuclear localization of a non-caspase truncation product of Atrophin-1, with an expanded polyglutamine repeat, increases cellular toxicity. J. Biol. Chem. 278, 13047–13055. 10.1074/jbc.M211224200 12464607

[B90] Ono Y. Saido T. C. Sorimachi H. (2016). Calpain research for drug discovery: challenges and potential. Nat. Rev. Drug. Discov. 15, 854–876. 10.1038/nrd.2016.212 27833121

[B89] Ono Y. Sorimachi H. (2012). Calpains: an elaborate proteolytic system. Biochim. Biophys. Acta 1824, 224–236. 10.1016/j.bbapap.2011.08.005 21864727

[B91] Orr H. T. (2012). Cell biology of spinocerebellar ataxia. J. Cell Biol. 197, 167–177. 10.1083/jcb.201105092 22508507 PMC3328388

[B92] Paoletti P. Vila I. Rifé M. Lizcano J. M. Alberch J. Ginés S. (2008). Dopaminergic and glutamatergic signaling crosstalk in Huntington’s disease neurodegeneration: the role of p25/cyclin-dependent kinase 5. J. Neurosci. 28, 10090–10101. 10.1523/JNEUROSCI.3237-08.2008 18829967 PMC6671267

[B93] Paulson H. L. Perez M. K. Trottier Y. Trojanowski J. Q. Subramony S. H. Das S. S.. (1997). Intranuclear inclusions of expanded polyglutamine protein in spinocerebellar ataxia type 3. Neuron 19, 333–344. 10.1016/s0896-6273(00)80943-5 9292723

[B94] Paulson H. L. Shakkottai V. G. Clark H. B. Orr H. T. (2017). Polyglutamine spinocerebellar ataxias - from genes to potential treatments. Nat. Rev. Neurosci. 18, 613–626. 10.1038/nrn.2017.92 28855740 PMC6420820

[B95] Ratovitski T. Chighladze E. Waldron E. Hirschhorn R. R. Ross C. a. (2011). Cysteine proteases bleomycin hydrolase and cathepsin Z mediate N-terminal proteolysis and toxicity of mutant huntingtin. J. Biol. Chem. 286, 12578–12589. 10.1074/jbc.M110.185348 21310951 PMC3069459

[B96] Ratovitski T. Nakamura M. D’Ambola J. Chighladze E. Liang Y. Wang W.. (2007). N-terminal proteolysis of full-length mutant huntingtin in an inducible PC12 cell model of Huntington’s disease. Cell Cycle 6, 2970–2981. 10.4161/cc.6.23.4992 18156806

[B97] Ravulapalli R. Campbell R. L. Gauthier S. Y. Dhe-Paganon S. Davies P. L. (2009). Distinguishing between calpain heterodimerization and homodimerization. FEBS J. 276, 973–982. 10.1111/j.1742-4658.2008.06833.x 19215300

[B98] Reijonen S. Kukkonen J. P. Hyrskyluoto A. Kivinen J. Kairisalo M. Takei N.. (2010). Downregulation of NF-κB signaling by mutant huntingtin proteins induces oxidative stress and cell death. Cell Mol. Life Sci. 67, 1929–1941. 10.1007/s00018-010-0305-y 20232225 PMC11115952

[B99] Richard I. Broux O. Allamand V. Fougerousse F. Chiannilkulchai N. Bourg N.. (1995). Mutations in the proteolytic enzyme calpain 3 cause limb-girdle muscular dystrophy type 2A. Cell 81, 27–40. 10.1016/0092-8674(95)90368-2 7720071

[B100] Robinson K. J. Yuan K. Plenderleith S. K. Watchon M. Laird A. S. (2021). A novel calpain inhibitor compound has protective effects on a zebrafish model of spinocerebellar ataxia type 3. Cells 10:2592. 10.3390/cells10102592 34685571 PMC8533844

[B101] Rogers L. D. Overall C. M. C. (2013). Proteolytic post-translational modification of proteins: proteomic tools and methodology. Mol. Cell. Proteomics 12:3532. 10.1074/mcp.M113.031310 23887885 PMC3861706

[B102] Sathasivam K. Neueder A. Gipson T. A. Landles C. Benjamin A. C. Bondulich M. K.. (2013). Aberrant splicing of HTT generates the pathogenic exon 1 protein in Huntington disease. Proc. Natl. Acad. Sci. U S A 110, 2366–2370. 10.1073/pnas.1221891110 23341618 PMC3568346

[B103] Schád E. Farkas A. Jékely G. Tompa P. Friedrich P. (2002). A novel human small subunit of calpains. Biochem. J. 362, 383–388. 10.1016/j.jep.2022.115747 11853546 PMC1222398

[B104] Schilling B. Gafni J. Torcassi C. Cong X. Row R. H. LaFevre-Bernt M. a.. (2006). Huntingtin phosphorylation sites mapped by mass spectrometry. Modulation of cleavage and toxicity. J. Biol. Chem. 281, 23686–23697. 10.1074/jbc.M513507200 16782707

[B105] Schilling G. Klevytska A. Tebbenkamp A. T. N. Juenemann K. Cooper J. Gonzales V.. (2007). Characterization of huntingtin pathologic fragments in human Huntington disease, transgenic mice and cell models. J. Neuropathol. Exp. Neurol. 66, 313–320. 10.1097/nen.0b013e318040b2c8 17413322

[B106] Schilling G. Wood J. D. Duan K. Slunt H. H. Gonzales V. Yamada M.. (1999). Nuclear accumulation of truncated atrophin-1 fragments in a transgenic mouse model of DRPLA. Neuron 24, 275–286. 10.1016/s0896-6273(00)80839-9 10677044

[B107] Selkoe D. J. (1994). Cell biology of the amyloid beta-protein precursor and the mechanism of Alzheimer’s disease. Annu. Rev. Cell Biol. 10, 373–403. 10.1146/annurev.cb.10.110194.002105 7888181

[B108] Selkoe D. J. Hardy J. (2016). The amyloid hypothesis of Alzheimer’s disease at 25 years. EMBO Mol. Med. 8, 595–608. 10.15252/emmm.201606210 27025652 PMC4888851

[B109] Simões A. T. Carmona V. Duarte-Neves J. Cunha-Santos J. Pereira de Almeida L. (2022). Identification of the calpain-generated toxic fragment of ataxin-3 protein provides new avenues for therapy of Machado-Joseph disease| Spinocerebellar ataxia type 3. Neuropathol. Appl. Neurobiol. 48:e12748. 10.1111/nan.12748 34273111

[B110] Simões A. T. Gonçalves N. Koeppen A. Déglon N. Kügler S. Duarte C. B.. (2012). Calpastatin-mediated inhibition of calpains in the mouse brain prevents mutant ataxin 3 proteolysis, nuclear localization and aggregation, relieving Machado-Joseph disease. Brain 135, 2428–2439. 10.1016/j.annemergmed.2022.04.016 22843411

[B111] Simões A. T. Gonçalves N. Nobre R. J. Duarte C. B. Pereira de Almeida L. (2014). Calpain inhibition reduces ataxin-3 cleavage alleviating neuropathology and motor impairments in mouse models of Machado-Joseph disease. Hum. Mol. Genet. 23, 4932–4944. 10.1016/j.annemergmed.2022.04.016 24817574

[B112] Smith M. A. Schnellmann R. G. (2012). Calpains, mitochondria and apoptosis. Cardiovasc. Res. 96, 32–37. 10.1093/cvr/cvs163 22581845 PMC3444233

[B113] Sorimachi H. Hata S. Ono Y. (2011). Calpain chronicle–an enzyme family under multidisciplinary characterization. Proc. Jpn. Acad. Ser. B Phys. Biol. Sci. 87, 287–327. 10.2183/pjab.87.287 21670566 PMC3153876

[B114] Sorimachi H. Imajoh-Ohmi S. Emori Y. Kawasaki H. Ohno S. Minami Y.. (1989). Molecular cloning of a novel mammalian calcium-dependent protease distinct from both m- and μ-types. J. Biol. Chem. 264, 20106–20111. 2555341

[B115] Sorimachi H. Ono Y. (2012). Regulation and physiological roles of the calpain system in muscular disorders. Cardiovasc. Res. 96, 11–22. 10.1093/cvr/cvs157 22542715 PMC3444232

[B116] Southwell A. L. Bugg C. W. Kaltenbach L. S. Dunn D. Butland S. Weiss A.. (2011). Perturbation with intrabodies reveals that calpain cleavage is required for degradation of huntingtin exon 1. PLoS One 6:e16676. 10.1371/journal.pone.0016676 21304966 PMC3031625

[B117] Spinozzi S. Albini S. Best H. Richard I. (2021). Calpains for dummies: what you need to know about the calpain family. Biochim. Biophys. Acta Proteins Proteom. 1869:140616. 10.1016/j.bbapap.2021.140616 33545367

[B118] Stoyas C. A. la Spada A. R. (2018). The CAG-polyglutamine repeat diseases: a clinical, molecular, genetic and pathophysiologic nosology. Handb. Clin. Neurol. 147, 143–170. 10.1016/B978-0-444-63233-3.00011-7 29325609

[B119] Suzuki Y. Nakayama K. Hashimoto N. Yazawa I. (2010). Proteolytic processing regulates pathological accumulation in dentatorubral-pallidoluysian atrophy. FEBS J. 277, 4873–4887. 10.1111/j.1742-4658.2010.07893.x 20977674

[B121] Tanaka Y. Igarashi S. Nakamura M. Gafni J. Torcassi C. Schilling G.. (2006). Progressive phenotype and nuclear accumulation of an amino-terminal cleavage fragment in a transgenic mouse model with inducible expression of full-length mutant huntingtin. Neurobiol. Dis. 21, 381–391. 10.1016/j.nbd.2005.07.014 16150600

[B120] Takano J. Mihira N. Fujioka R. Hosoki E. Chishti A. H. Saido T. C. (2011). Vital role of the calpain-calpastatin system for placental-integrity-dependent embryonic survival. Mol. Cell Biol. 31, 4097–4106. 10.1128/MCB.05189-11 21791606 PMC3187362

[B122] Tompa P. Emori Y. Sorimachi H. Suzuki K. Friedrich P. (2001). Domain III of calpain is a Ca^2+^-regulated phospholipid-binding domain. Biochem. Biophys. Res. Commun. 280, 1333–1339. 10.1006/bbrc.2001.4279 11162675

[B123] Toonen L. J. A. Schmidt I. Luijsterburg M. S. van Attikum H. van Roon-Mom W. M. C. (2016). Antisense oligonucleotide-mediated exon skipping as a strategy to reduce proteolytic cleavage of ataxin-3. Sci. Rep. 6:35200. 10.1038/srep35200 27731380 PMC5059676

[B124] Vissing J. Barresi R. Witting N. Van Ghelue M. Gammelgaard L. Bindoff L. A.. (2016). A heterozygous 21-bp deletion in CAPN3 causes dominantly inherited limb girdle muscular dystrophy. Brain 139, 2154–2163. 10.1093/brain/aww133 27259757

[B125] Wang Y. Bi X. Baudry M. (2018). Calpain-2 as a therapeutic target for acute neuronal injury. Expert Opin. Ther. Targets 22, 19–29. 10.1080/14728222.2018.1409723 29168923 PMC6211856

[B126] Wang Y. Hersheson J. Lopez D. Hammer M. Liu Y. Lee K.-H.. (2016). Defects in the CAPN1 gene result in alterations in cerebellar development and cerebellar ataxia in mice and humans. Cell Rep. 16, 79–91. 10.1016/j.celrep.2016.05.044 27320912 PMC4927383

[B127] Watchon M. Yuan K. C. Mackovski N. Svahn A. J. Cole N. J. Goldsbury C.. (2017). Calpain inhibition is protective in machado-joseph disease zebrafish due to induction of autophagy. J. Neurosci. 37, 7782–7794. 10.1523/JNEUROSCI.1142-17.2017 28687604 PMC6596655

[B128] Weber J. J. Anger S. C. Pereira Sena P. Incebacak Eltemur R. D. Huridou C. Fath F.. (2022). Calpains as novel players in the molecular pathogenesis of spinocerebellar ataxia type 17. Cell. Mol. Life Sci. 79:262. 10.1007/s00018-022-04274-6 35482253 PMC9050766

[B129] Weber J. J. Clemensson L. E. Schiöth H. B. Nguyen H. P. (2019b). Olesoxime in neurodegenerative diseases: scrutinising a promising drug candidate. Biochem. Pharmacol. 168, 305–318. 10.1016/j.bcp.2019.07.002 31283931

[B130] Weber J. J. Golla M. Guaitoli G. Wanichawan P. Hayer S. N. Hauser S.. (2017). A combinatorial approach to identify calpain cleavage sites in the Machado-Joseph disease protein ataxin-3. Brain 140, 1280–1299. 10.1093/brain/awx039 28334907

[B131] Weber J. J. Haas E. Maringer Y. Hauser S. Casadei N. L. P. Chishti A. H.. (2020). Calpain-1 ablation partially rescues disease-associated hallmarks in models of Machado-Joseph disease. Hum. Mol. Genet. 29, 892–906. 10.1093/hmg/ddaa010 31960910 PMC7158375

[B132] Weber J. J. Kloock S. J. Nagel M. Ortiz-Rios M. M. Hofmann J. Riess O.. (2018). Calpastatin ablation aggravates the molecular phenotype in cell and animal models of Huntington disease. Neuropharmacology 133, 94–106. 10.1016/j.neuropharm.2018.01.022 29355642

[B133] Weber J. J. Ortiz Rios M. M. Riess O. Clemens L. E. Nguyen H. P. (2016). The calpain-suppressing effects of olesoxime in Huntington’s disease. Rare Dis. 4:e1153778. 10.1080/21675511.2016.1153778 27141414 PMC4838320

[B134] Weber J. J. Pereira Sena P. Singer E. Nguyen H. P. (2019a). Killing two angry birds with one stone: autophagy activation by inhibiting calpains in neurodegenerative diseases and beyond. Biomed. Res. Int. 2019:4741252. 10.1155/2019/4741252 30895192 PMC6393885

[B135] Weber J. J. Sowa A. S. Binder T. Hübener J. (2014). From pathways to targets: understanding the mechanisms behind polyglutamine disease. Biomed. Res. Int. 2014:701758. 10.1155/2014/701758 25309920 PMC4189765

[B137] Wellington C. L. Ellerby L. M. Gutekunst C.-A. Rogers D. Warby S. Graham R. K.. (2002). Caspase cleavage of mutant huntingtin precedes neurodegeneration in Huntington’s disease. J. Neurosci. 22, 7862–7872. 10.1523/JNEUROSCI.22-18-07862.2002 12223539 PMC6758089

[B138] Wellington C. L. Ellerby L. M. Hackam A. S. Margolis R. L. Trifiro M. a. Singaraja R.. (1998). Caspase cleavage of gene products associated with triplet expansion disorders generates truncated fragments containing the polyglutamine tract. J. Biol. Chem. 273, 9158–9167. 10.1074/jbc.273.15.9158 9535906

[B136] Wellington C. L. Hayden M. R. (1997). Of molecular interactions, mice and mechanisms: new insights into Huntington’s disease. Curr. Opin. Neurol. 10, 291–298. 10.1097/00019052-199708000-00003 9266152

[B139] Wellington C. L. Singaraja R. Ellerby L. Savill J. Roy S. Leavitt B.. (2000). Inhibiting caspase cleavage of huntingtin reduces toxicity and aggregate formation in neuronal and nonneuronal cells. J. Biol. Chem. 275, 19831–19838. 10.1074/jbc.M001475200 10770929

[B140] Wright A. L. Vissel B. (2016). CAST your vote: is calpain inhibition the answer to ALS? J. Neurochem. 137, 140–141. 10.1111/jnc.13296 27005822

[B141] Yang H. Murthy S. Sarkar F. H. Sheng S. Reddy G. P. V. Dou Q. P. (2008). Calpain-mediated androgen receptor breakdown in apoptotic prostate cancer cells. J. Cell. Physiol. 217, 569–576. 10.1002/jcp.21565 18726991 PMC2597227

[B142] Young J. E. Gouw L. Propp S. Sopher B. L. Taylor J. Lin A.. (2007). Proteolytic cleavage of ataxin-7 by caspase-7 modulates cellular toxicity and transcriptional dysregulation. J. Biol. Chem. 282, 30150–30160. 10.1074/jbc.M705265200 17646170

[B143] Yousefi S. Perozzo R. Schmid I. Ziemiecki A. Schaffner T. Scapozza L.. (2006). Calpain-mediated cleavage of Atg5 switches autophagy to apoptosis. Nat. Cell Biol. 8, 1124–1132. 10.1038/ncb1482 16998475

[B144] Zha C. Farah C. A. Holt R. J. Ceroni F. Al-Abdi L. Thuriot F.. (2020). Biallelic variants in the small optic lobe calpain CAPN15 are associated with congenital eye anomalies, deafness and other neurodevelopmental deficits. Hum. Mol. Genet. 29, 3054–3063. 10.1093/hmg/ddaa198 32885237 PMC7645705

[B145] Zhou H. Cao F. Wang Z. Yu Z. X. Nguyen H. P. Evans J.. (2003). Huntingtin forms toxic NH2-terminal fragment complexes that are promoted by the age-dependent decrease in proteasome activity. J. Cell Biol. 163:109. 10.1083/jcb.200306038 14557250 PMC2173440

[B146] Zimmerman U.-J. P. Boring L. Pak J. H. Mukerjee N. Wang K. K. W. (2000). The calpain small subunit gene is essential: its inactivation results in embryonic lethality. IUBMB Life 50, 63–68. 10.1080/15216540050176610 11087123

